# Phase 2a Study of Ataluren-Mediated Dystrophin Production in Patients with Nonsense Mutation Duchenne Muscular Dystrophy

**DOI:** 10.1371/journal.pone.0081302

**Published:** 2013-12-11

**Authors:** Richard S. Finkel, Kevin M. Flanigan, Brenda Wong, Carsten Bönnemann, Jacinda Sampson, H. Lee Sweeney, Allen Reha, Valerie J. Northcutt, Gary Elfring, Jay Barth, Stuart W. Peltz

**Affiliations:** 1 Children’s Hospital of Philadelphia, Philadelphia, Pennsylvania, United States of America; 2 Nemours Children’s Hospital, Orlando, Florida, United States of America; 3 University of Utah School of Medicine, Salt Lake City, Utah, United States of America; 4 Cincinnati Children’s Hospital Medical Center, Cincinnati, Ohio, United States of America; 5 Perelman School of Medicine at the University of Pennsylvania, Philadelphia, Pennsylvania, United States of America; 6 PTC Therapeutics, Inc., South Plainfield, New Jersey, United States of America; National Hospital of Utano, Japan

## Abstract

**Background:**

Approximately 13% of boys with Duchenne muscular dystrophy (DMD) have a nonsense mutation in the dystrophin gene, resulting in a premature stop codon in the corresponding mRNA and failure to generate a functional protein. Ataluren (PTC124) enables ribosomal readthrough of premature stop codons, leading to production of full-length, functional proteins.

**Methods:**

This Phase 2a open-label, sequential dose-ranging trial recruited 38 boys with nonsense mutation DMD. The first cohort (n = 6) received ataluren three times per day at morning, midday, and evening doses of 4, 4, and 8 mg/kg; the second cohort (n = 20) was dosed at 10, 10, 20 mg/kg; and the third cohort (n = 12) was dosed at 20, 20, 40 mg/kg. Treatment duration was 28 days. Change in full-length dystrophin expression, as assessed by immunostaining in pre- and post-treatment muscle biopsy specimens, was the primary endpoint.

**Findings:**

Twenty three of 38 (61%) subjects demonstrated increases in post-treatment dystrophin expression in a quantitative analysis assessing the ratio of dystrophin/spectrin. A qualitative analysis also showed positive changes in dystrophin expression. Expression was not associated with nonsense mutation type or exon location. Ataluren trough plasma concentrations active in the *mdx* mouse model were consistently achieved at the mid- and high- dose levels in participants. Ataluren was generally well tolerated.

**Interpretation:**

Ataluren showed activity and safety in this short-term study, supporting evaluation of ataluren 10, 10, 20 mg/kg and 20, 20, 40 mg/kg in a Phase 2b, double-blind, long-term study in nonsense mutation DMD.

**Trial Registration:**

ClinicalTrials.gov NCT00264888

## Introduction

Duchenne muscular dystrophy (DMD) results from mutations in the gene encoding dystrophin, a protein that stabilizes muscle cell membranes. The absence of normally functioning dystrophin results in contraction-induced membrane injury. Patients with DMD develop progressive proximal muscle weakness that leads to deterioration of ambulation, wheelchair dependency, and eventual respiratory and cardiac failure. Pharmacotherapy is limited to corticosteroids, which increase muscle strength in the short-term but have significant side effects and do not address the underlying cause of DMD. Strategies in clinical trials to restore dystrophin in muscle cell membranes include nonsense mutation suppression and exon skipping [1].

A nonsense mutation is a single-point alteration in DNA that results in the inappropriate presence of a UAA, UAG, or UGA stop codon in the protein-coding region of the corresponding mRNA. This premature stop codon causes the production of a truncated protein and leads to loss of protein function and consequent disease. Nonsense mutations are responsible for approximately 13% of DMD cases [2].

Ataluren (also known as PTC124) was discovered through high-throughput screening and chemical optimization to induce ribosomes to read through premature stop codons but not normal stop codons. [3] When tested in the nonsense mutation *mdx* mouse model of DMD, ataluren generated production of full-length, functional dystrophin protein. [3], [4] We and others have shown that ataluren is active in multiple cell-based and animal disease models.[3]–[9] Phase 1 studies in healthy volunteers established the initial ataluren safety profile [10] and defined dosing regimens for achieving target trough plasma concentrations (2 to 10 µg/mL) known to be active in preclinical models [3], [5].

We describe here the results from a Phase 2a clinical trial in subjects with nonsense mutation DMD that evaluated pharmacodynamic activity, as measured by immunofluorescence evidence of an increase in dystrophin production on muscle biopsy. The study also assessed additional markers of disease activity, changes in muscle strength and function, safety, and ataluren pharmacokinetics.

## Methods

The protocol for this trial and supporting CONSORT checklist are available as supporting information; see Checklist S1 and Protocol S1.

### Ethics

This study was performed at three sites (Children’s Hospital of Philadelphia, Cincinnati Children’s Hospital Medical Center, University of Utah School of Medicine) and was approved by each investigator’s institutional review board (IRB). The IRBs for this study were the Children’s Hospital of Philadelphia Committees for Protection of Human Subjects, Cincinnati Children’s Research Foundation, University of Utah, and the Western Institutional Review Board.

The trial was conducted in accordance with the Declaration of Helsinki and Good Clinical Practice and was registered (Identifier NCT00264888) at www.clinicaltrials.gov. A parent or guardian of each subject was required to sign an informed consent form approved by the local IRB. Subjects were also required to provide signed assent to participate.

### Eligibility Criteria

Since DMD is an X-linked disease, only male subjects were enrolled. Subjects were ≥5 years of age and were required to have a diagnosis of nonsense mutation DMD based on a clinical phenotype present by age 5, increased serum CK, absent or diminished sarcolemmal staining with an antibody to the C-terminal portion of the dystrophin protein on muscle biopsy, and presence of a nonsense mutation in the dystrophin gene (as confirmed by gene sequencing). Complete entry criteria are provided in the Appendix S1.

### Study Treatment

Eligible subjects were sequentially assigned to escalating dose levels of ataluren. The first patient was recruited on 21 December, 2005, and follow-up was completed on 03 May, 2007. Subjects were not to be enrolled at the next higher ataluren dose level until all subjects who had been treated at the previous level had completed the 28-day treatment period and a review of safety and pharmacokinetic data had indicated that dose escalation was appropriate. The study was originally planned to include two dose groups. The first (N = 6) and second (N = 20) groups of subjects received treatment with an oral suspension of ataluren TID at morning, midday, and evening doses of 4, 4, 8 mg/kg and 10, 10, 20 mg/kg, respectively. Both dose levels were well tolerated. Based on pharmacokinetic data at these dose levels, the protocol was amended and an additional 12 subjects were enrolled to receive ataluren 20, 20, 40 mg/kg. The duration of treatment was 28 days. Subjects were followed for an additional 28 days post-treatment.

### Study Assessments

Excisional biopsies of the extensor digitorum brevis (EDB) muscles were performed on all subjects [11]; the muscle was obtained from one foot at baseline and the other foot on Day 28 of treatment. Immunofluorescence analysis of serial 9-µm cross sections was performed as the primary outcome measure. Immunofluorescence images were analyzed qualitatively and quantitatively, as detailed in the Appendix S1. In addition, myotubes were derived from baseline EDB biopsy specimens and cultured in vitro in the presence of a range of ataluren concentrations, including 10 µg/mL. Western blot analysis of changes in dystrophin and other muscle membrane proteins was not performed. Serum CK levels were monitored at baseline, every seven days during treatment, and at Days 14 and 28 post-treatment. Myometry and timed function tests were performed at baseline, after 28 days of treatment, and Day 28 post-treatment.

Blood samples for ataluren pharmacokinetic assessments were collected pre-dose and 1, 2, 3, and 4 hours after the morning dose; pre-dose and 1, 2, 3, and 4 hours after the midday dose; and pre-dose and 1, 2, 3, 4, and 12 hours after the evening dose. Ataluren plasma concentrations were derived from a validated bioanalytical method [10].

### Safety Monitoring

Safety monitoring included adverse events, laboratory tests, vital signs, electrocardiograms, and physical examinations. An independent data safety monitoring board, comprising two neuromuscular experts and a biostatistician, reviewed safety data during the study.

### Statistics

All subjects were included in safety and compliance analyses. Subjects with both baseline and on-study measurements were included in efficacy analyses. The number and percentage of subjects with a post-treatment dystrophin response in muscle biopsy were computed for each dose group. The number and percentage of subjects who had an in vitro ataluren dystrophin response were computed. Changes in quantitative dystrophin expression, serum CK, myometry, and timed function tests were analyzed using paired *t*-tests. Pretreatment serum CK values were obtained at screening and baseline; the mean of each subject’s screening and baseline values was used for analyses. Compliance was calculated as the proportion of actual relative to planned ataluren doses. Pharmacokinetic parameters were calculated using non-compartmental methods.

The term, “percentage increase,” utilized in this paper represents an increase relative to baseline in light intensity.

The statistical software used for the analyses was Statistical Analysis System (SAS) version 9.1.

## Results

### Subjects

**Figure 1:**
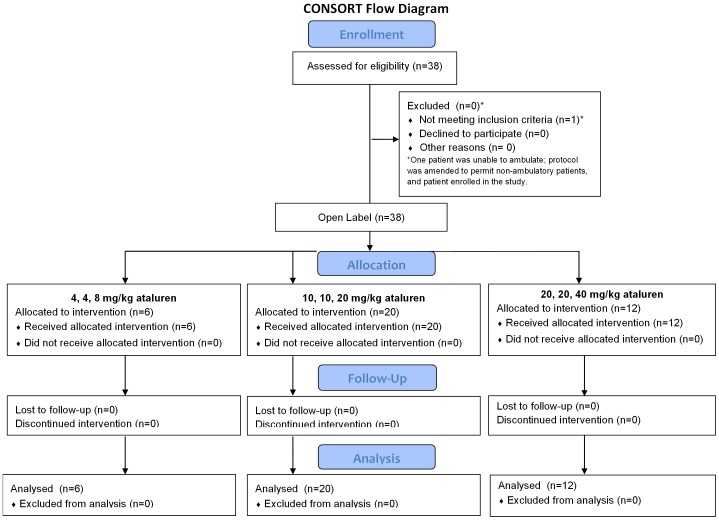
CONSORT Flow Diagram.

Thirty-eight subjects were enrolled and completed the study. By dose level, six subjects received 4, 4, 8 mg/kg; 20 subjects received 10, 10, 20 mg/kg; and 12 subjects received 20, 20, 40 mg/kg (Figure 1).

Ages and body weights were generally consistent across the dose groups, albeit with a higher range at the 20, 20, 40 mg/kg dose level due to inclusion of several older, nonambulatory boys in this cohort (Table 1). Of the 38 subjects, 34 (89·5%) were ambulatory as determined by the investigators. Because of generalized muscle fragility in DMD, serum CK and transaminase values were universally abnormal at baseline. Twenty-seven of 38 subjects (71·1%) were receiving corticosteroid treatment, usually with a daily regimen of deflazacort or prednisone/prednisolone. All 3 types of premature stop codons were represented, but the UGA stop codon was predominant in all dose groups. Nonsense mutations were located across exons 6 to 70 of the dystrophin gene; no mutational hotspots (ie, specific locations with notably higher numbers of mutations) were identified.

**Table 1:** Baseline Subject Characteristics.

	Ataluren Dose Groups		
	4, 4, 8 mg/kg	10, 10, 20 mg/kg	20, 20, 40 mg/kg
Characteristic	(N = 6)	(N = 20)	(N = 12)
Age (years)			
n	6	20	12
Mean ± SD	8·3±2·34	8·5±1·70	9·6±3·65
Median	9·0	8·5	9·0
Range	5–11	6–12	5–17
Sex – n (%)			
Male	6 (100·0)	20 (100·0)	12 (100·0)
Female	0 (0·0)	0 (0·0)	0 (0·0)
Race – n (%)			
Caucasian	6 (100·0)	15 (75·0)	11 (91·7)
Black	0 (0·0)	0 (0·0)	0 (0·0)
Asian	0 (0·0)	3 (15·0)	0 (0·0)
Hispanic	0 (0·0)	0 (0·0)	0 (0·0)
Other	0 (0·0)	2 (10·0)	1 (8·3)
Weight			
n	6	20	12
Mean ± SD	29·33±9·895	31·25±10·164	31·95±17·893
Median	30·50	28.95	24·25
Range	17·3–39·7	18·5–57·5	17·5–74·2
Ability to ambulate – n(%)^1^			
No	0 (0·0)	1 (5·0)	3 (25·0)
Yes	6 (100·0)	19 (95·0)	9 (75·0)
Premature stop codon type – n(%)			
UGA	4 (66·7)	11 (55·0)	7 (58·3)
UAG	2 (33·3)	5 (25·0)	1 (8·3)
UAA	0 (0·0)	4 (20·0)	4 (33·3)
Location of mutations on dystrophin gene– range of exon numbers	24 to 70	6 to 70	6 to 61

1 Ability to ambulate was based on investigator’s judgment.

**Abbreviations**: SD = SD = standard deviation, UAA = uridine-adenosine-adenosine, UAG = uridine adenosine guanosine, UGA = uridine-guanosine-adenosine.

### Changes in Muscle Dystrophin Expression in Muscle Myotubes Cultured in vitro

Pre-treatment primary muscle cells from 35 of the 38 subjects were available for in vitro myotube culture. Myoblasts were expanded in growth medium, transferred to differentiation medium, and allowed to progress for three days. Differentiated myotubes were subsequently exposed to ataluren 10 µg/ml for nine days prior to dystrophin staining. Dystrophin levels were analyzed by quantitative immunofluoresence. The results demonstrate that when the muscle obtained from the pre-treatment biopsies were cultured in the presence of ataluren, 35/35 (100%) of the samples showed evidence of an increase in dystrophin expression in response to ataluren treatment in vitro (Table 2).

**Table 2:** In Vivo and In Vitro Dystrophin Expression.

							Dystrophin Expression Response to Treatment		
Site No.	Subject No.	Age (years)	Steroid Use	Mutation	Exon	Stop Codon	In Vitro^b^	In Vivo^a^	
								Qualitative	Quantitative
								Ataluren 4, 4, 8 mg/kg (N = 6)	
001	001	11	Yes	W1268X	28	UGA	10·8	Yes	17.78%
001	002	10	Yes	E2035X	42	UAG	12·2	Yes	26.17%
002	001	9	Yes	S3127X	65	UGA	4·8	No	22.79%
002	002	9	Yes	W1075X	24	UAG	7·9	No	−14.24%
003	001	6	Yes	R3381X	70	UGA	ND^c^	No	−1.57%
003	002	5	Yes	R3034X	61	UGA	ND^c^	No	22.94%
*Total for Ataluren 4, 4, 8 mg/kg*								*Yes: 2/6 (33%)*	*Mean: 12.31% Yes: 4/6 (67%)*
								**Ataluren 10, 10, 20 mg/kg (N = 20)**	
001	003	10	No	E2286X	47	UAA	31·6	No	−22.08%
001	004	6	No	E2035X	42	UAG	10·5	Yes	−6.5%
001	005	10	Yes	E1182X	26	UAG	10·1	No	12.02%
001	006	9	No	R3391X	70	UGA	15·0	No	0.35%
001	007	9	Yes	Q1885X	40	UGA	9·9	Yes	−1.97%
001	008	9	Yes	Q2574X	53	UAG	17·2	Yes	18.2%
001	009	8	No	E2894X	59	UGA	2·2	Yes	39.26%
001	010	8	Yes	R145X	6	UGA	5·8	Yes	−0.2%
002	003	9	Yes	W1879X	40	UGA	3·9	No	16.5%
002	004	13	Yes	R1844X	39	UGA	13·7	Yes	56.47%
002	005	11	Yes	Q555X	14	UAA	1·4	No	28.32%
002	006	8	Yes	W2925X	59	UGA	18·3	No	−25.53%
002	007	8	Yes	W1956X	41	UAG	16·7	No	−3.17%
002	008	7	Yes	Y1882X	40	UAA	2·8	Yes	−9.9%
003	003	8	Yes	R539X	14	UGA	3·3	No	22.31%
003	004	7	No	Q194X	7	UAA	3·3	No	13.86%
003	005	7	Yes	R145X	6	UGA	8·0	No	4.56%
003	006	12	Yes	R1967X	41	UGA	2·2	No	−12.71%
003	007	7	No	K871X	20	UGA	4·3	No	−0.46%
003	008	11	No	Q267X	8	UAG	18·9	Yes	39.07%
*Total for Ataluren 10, 10, 20 mg/kg*								*Yes: 8/20 (40%)*	*Mean:8.42% Yes: 11/20 (55%)*
								**Ataluren 20, 20, 40 mg/kg (N = 12)**	
001	011	6	Yes	Q2526X	52	UAG	7·7	Yes	14.59%
001	012	8	Yes	R2905X	59	UGA	12·8	No	−0.28%
001	013	8	Yes	R3034X	61	UGA	10·1	No	5.45%
001	014	10	No	Q555X	14	UAA	1·8	No	14%
001	015	5	No	Q555X	14	UAA	11·9	No	−23.12%
001	016	9	Yes	K2791X	56	UAA	16·1	Yes	95.14%
002	009	17	Yes	R2870X	58	UGA	23·0	Yes	39.80%
002	010	14	No	L654X	16	UAA	20·3	No	−1.91%
002	011	14	No	R1967X	41	UGA	ND^d^	No	36.38%
002	012	6	Yes	S147X	6	UGA	5·5	No	−29.85%
003	009	9	Yes	R3034X	61	UGA	10·7	No	18.55%
003	010	9	Yes	R195X	7	UGA	7·9	No	7.25%
*Total for Ataluren 20, 20, 40 mg/kg*								*Yes: 3/12 (25%)*	*Mean: 14.67% Yes: 9/12 (67%)*
*Total for All Dose Levels Combined*								*Yes: 13/38 (38%)*	*Mean: 11.0% Yes: 23/38 (61%)*

a Qualitative: Response = ≥2/3 blinded reviewers observed more dystrophin in post-treatment image compared to pre-treatment image. Quantitative: Change in dystrophin:spectrin ratio from pre-treatment to post-treatment.

b Fold change at 10 µg/mL over 0 µg/mL.

c Biopsies lost during transportation.

d Cells not viable in culture.

**Abbreviations**: ND = not done.

### In vivo Changes in Muscle Dystrophin Expression

**Figure 2:**
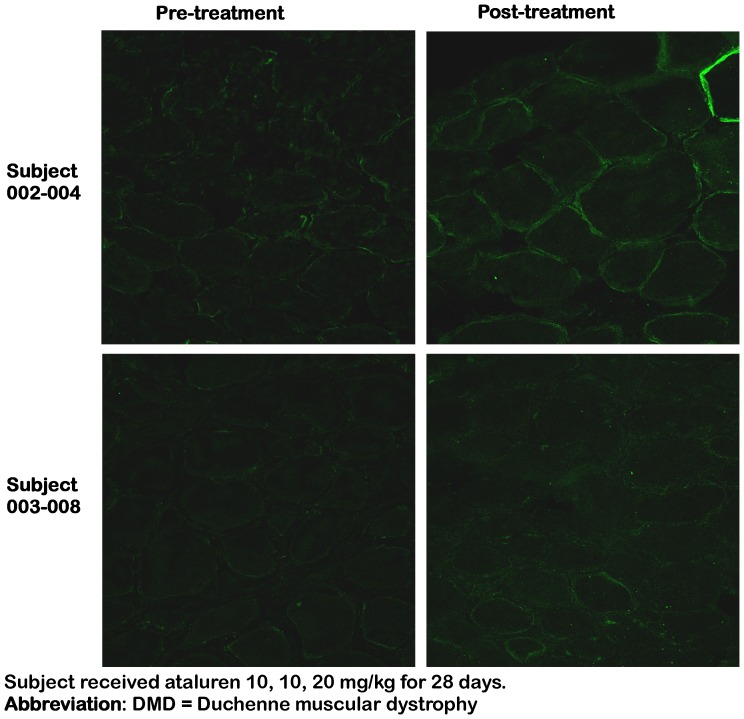
In Vivo Dystrophin Expression by Immunofluorescence in Extensor Digitorum Brevis Muscle: Example of a Responder Subject.

Pre- and post-treatment immunofluorescence images from the EDB muscles were available from all 38 subjects. These images were assessed qualitatively by blinded reviewers who determined whether dystrophin expression was higher in the pre-treatment image, the post-treatment image, unchanged, or indeterminate. At all three dose levels, subjects demonstrated qualitative increases in the staining for dystrophin, ie, at least 2 of 3 reviewers observed higher dystrophin expression in the post-treatment image (Table 2). Overall, 2/6 (33%) subjects treated at 4, 4, 8 mg/kg, 8/20 (40%) subjects treated at 10, 10, 20 mg/kg, and 3/12 (25%) subjects treated at 20, 20, 40 mg/kg demonstrated an increase in dystrophin expression post-treatment. Figure 2 provides examples of immunofluorescence data from two subjects who were considered responders by the evaluators, and Figure S1 is representative of a non-responder. Post-treatment dystrophin expression is appropriately localized in the cell membranes of the muscle fibers.

**Figure 3:**
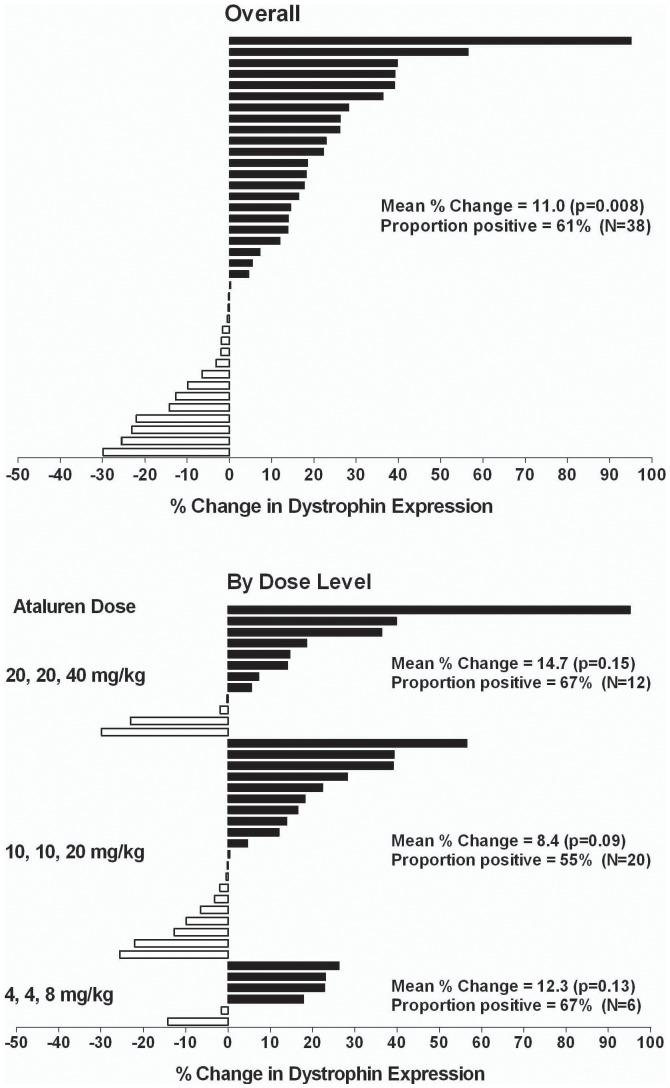
Percentage Change From Pretreatment in Dystrophin:Spectrin Ratio.

A quantitative method for assessing the ratio of dystrophin/spectrin expression was developed and became available for this study. Based on this quantitative analysis, a mean change from pretreatment to posttreatment of 11.0% in dystrophin expression was observed (p = 0.008, paired t-test). Of the 38 patients, 23 (61%) showed a positive change in dystrophin/spectrin expression ratio after 28 days of treatment with ataluren (Table 2, Figure 3).

Response did not appear to be dependent on age, corticosteroid use, or location or type of nonsense mutation in either method.

### Changes in Serum CK Levels

**Figure 4:**
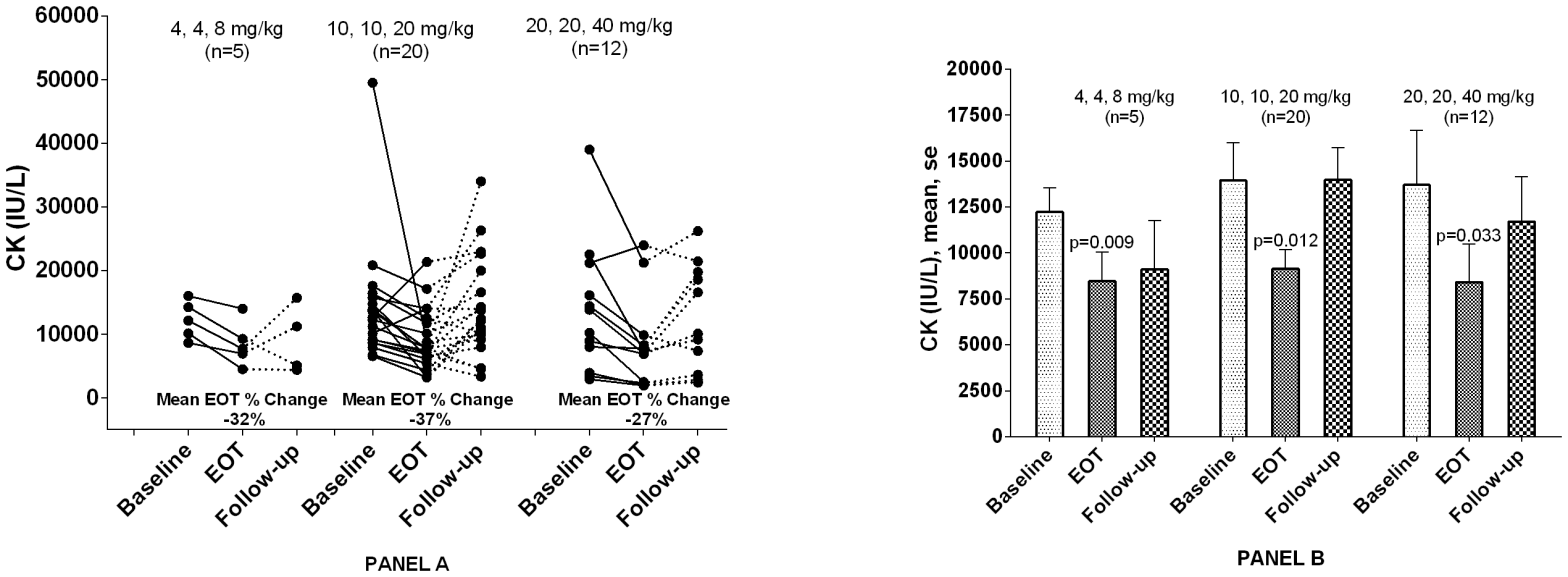
Serum Creatine Kinase Concentrations. Serum CK levels at baseline (mean of screening and Day 1 pretreatment), end-of-treatment (Day 28), and follow-up (Day 56). P-values obtained from paired t-tests. **Abbreviations:** CK = creatine kinase, EOT = end of treatment, nmDMD = nonsense mutation Duchenne muscular dystrophy, SEM = standard error of the mean.

Due to muscle fragility, serum CK concentrations are universally elevated in subjects with DMD. [12], [13] As shown in Figure 4, the majority of subjects in each cohort had decreases in serum CK values when comparing end-of-treatment values to pretreatment values. Although no definite dose-response relationship can be discerned due to small and varying sample sizes, these changes were statistically significant at the 10, 10, 20****mg/kg and 20, 20, 40 mg/kg dose levels, but not at the 4, 4, 8 mg/kg dose level. The return of mean values toward baseline upon cessation of treatment may provide further support for pharmacological activity.

### Changes in Clinical Measures

Collective changes in upper and/or lower extremity myometry scores (for hand grip, elbow flexion, hip abduction, and knee extension) and timed function tests (standing from supine, running 10 meters, climbing four standard stairs) were small and not statistically significant after 28 days of ataluren treatment.

Although a formal symptom survey was not used, parents and teachers of several boys anecdotally reported evidence of greater activity, increased endurance, and less fatigue during treatment.

### Safety

Adverse events were mild or moderate and showed no dose-dependent increase in frequency or severity. Procedural complications as a result of the muscle biopsy procedures (reported in 29 of the 38 subjects, 76·3%) represented the most frequently reported adverse events, followed by gastrointestinal-related events such as flatulence, diarrhea, vomiting, abdominal discomfort or pain, and nausea (reported in 22 of the 38 subjects, 57·9%).

No clinically significant, treatment-emergent laboratory abnormalities were observed.

One subject receiving the 20, 20, 40 mg/kg dose level experienced mild abdominal discomfort which resulted in the interruption of ataluren treatment on Day 3 to 4. The subject resumed ataluren at 15, 15, 30****mg/kg on Day 5 (upon resolution of the adverse events) and was re-escalated to 20, 20, 40 mg/kg dose level on Day 7; this dose level was well tolerated and the subject completed the study. No subject discontinued ataluren due to a drug-related adverse event.

### Pharmacokinetics

**Figure 5:**
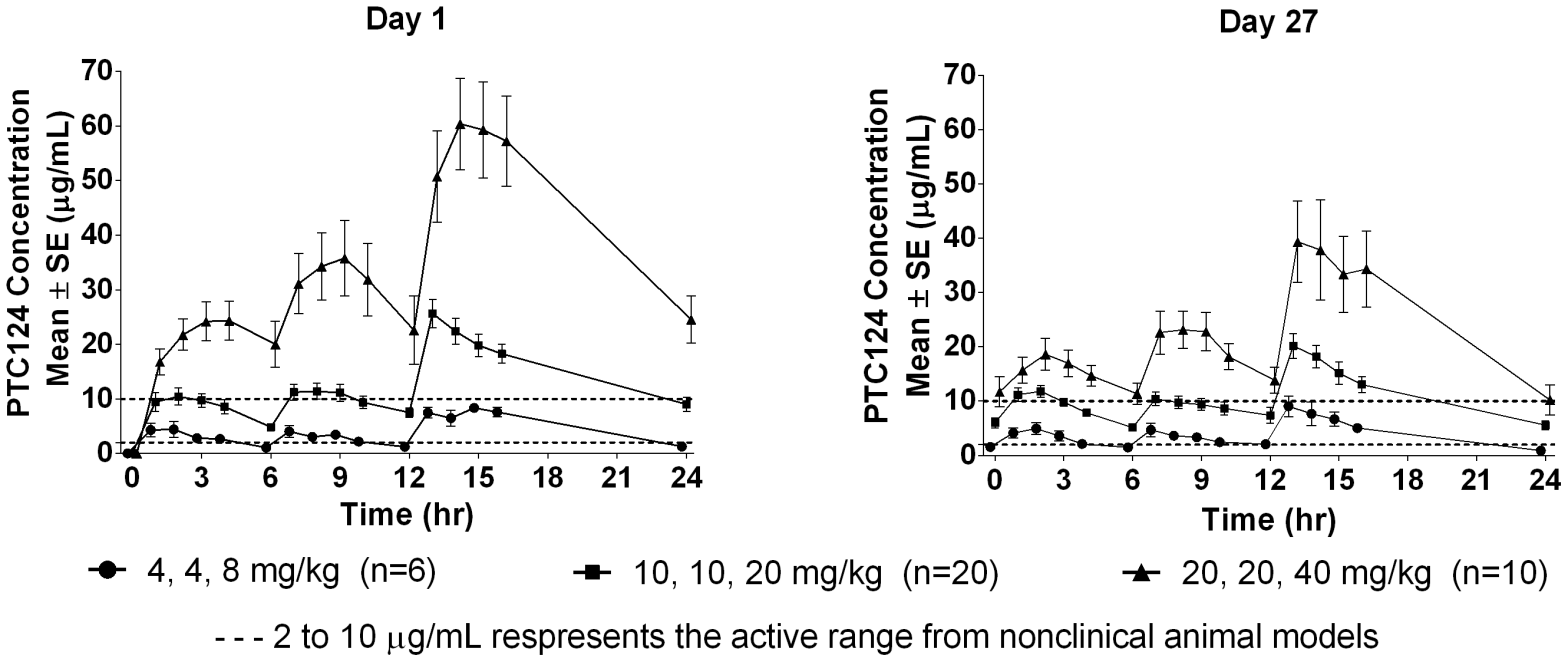
Ataluren Plasma Concentrations Over Time. Blood samples were collected pre-dose and at 1, 2, 3, and 4 hours after the morning dose; pre-dose and at 1, 2, 3, and 4 hours after the midday dose; and pre-dose and at 1, 2, 3, 4, and 12 hours after the evening dose. Ataluren plasma concentrations were derived from a validated bioanalytical method. **Abbreviations:** SE = standard error.

Ataluren was rapidly absorbed, with median T_max_ values after the morning dose ranging from 1·5 to 2·5 hours. The 10, 10, 20 mg/kg and 20, 20, 40 mg/kg dose levels produced trough plasma concentrations of ataluren that were in or above the range known to be active in nonclinical animal models (∼2 to ∼10 µg/mL); high intersubject variability and substantial overlap between these two dose groups was observed. Trough plasma concentrations for the 4, 4, 8 mg/kg dose level were near the lower end of the range and did not consistently produce levels that were in the target range (Figure 5). The 24-hour concentration of one subject in the 20, 20, 40 mg/kg dose group was excluded from all pharmacokinetic analyses because his value at the 24-hour timepoint on Day 1 (171 µg/mL) was as an outlier based on the Grubbs statistical outlier test [14].

**Figure 6:**
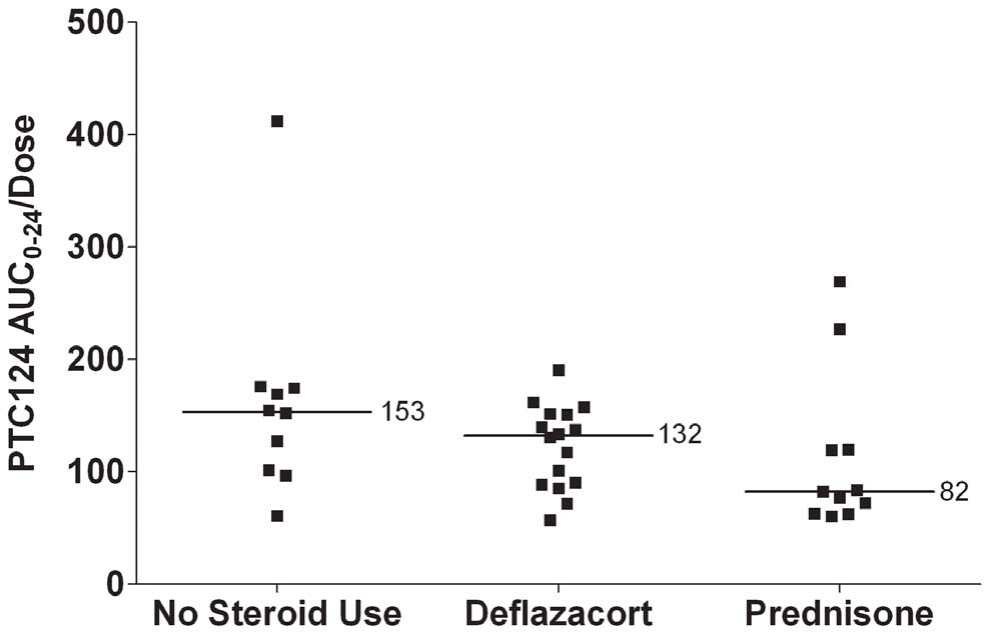
Dose-Normalized Day 27 AUC_0–24_ Ataluren Values by Concomitant Corticosteroid Use (All Dose Levels). Note: Subject 001–012 in the 20, 20, 40 mg/kg dose group was receiving prednisolone, a metabolite of prednisone. This subject is included in the prednisone group above. **Abbreviation:** AUC_0–24_ = area under the plasma concentration-time curve from 0 to 24 hours.

The potential effect of concomitant corticosteroid use on the pharmacokinetics of ataluren was investigated by stratifying the subjects by corticosteroid use (either prednisone/prednisolone, deflazacort, or no corticosteroid) and comparing dose-normalized area under the concentration-time curve through 24 hours (AUC_0–24_) values on Day 27 (Figure 6). Significant overlap was observed among the dose groups, with similar mean AUC_0–24_ values, suggesting the absence of a significant effect of corticosteroids on the pharmacokinetics of ataluren. While the data analysis indicates that there is no apparent effect of corticosteroid use on ataluren pharmacokinetics, the small sample size must be considered in interpretation of these data.

## Discussion

This Phase 2a, short-term study conducted from Dec 2005 to May 2007 evaluated whether ataluren, a first-in-class investigational new drug, can restore dystrophin production in the muscle cells of patients with DMD whose disease is caused by a nonsense mutation. This therapeutic approach holds considerable promise for such patients, who currently lack dystrophin-targeted, disease-modifying treatment options. Overall, 34% (13/38) of the subjects in this study showed evidence of an increase in dystrophin production on EDB muscle biopsy by immunofluorescence, as assessed qualitatively by independent reviewers who were blinded to timepoint and dose level. Correct localization of post-treatment dystrophin at the sarcolemma was observed. Revertant fibers were excluded from the dystrophin analysis. When analyzed quantitatively, 61% (23/38) of the subjects showed a post-treatment increase in dystrophin. These data documented pharmacodynamic proof-of-concept for ataluren in DMD and supported the conduct of a now-completed Phase 2b randomized, placebo-controlled, multiple dose, long-term efficacy and safety study (conducted from Feb 2008 to Dec 2009;ClinicalTrials.gov, NCT00592553), and for the confirmatory Phase 3 randomized placebo-controlled study (2013-ongoing; ClinicalTrials.gov, NCT01826487). Increases in in vivo dystrophin expression were not dose-dependent or correlated with demographic or genetic factors. It is unclear why some of the subjects showed increases in dystrophin production while others did not. Among other factors, the limited duration of drug exposure may account for the variability in the findings. Pharmacokinetic data showed that although there was overlap of the ataluren plasma concentrations substantially among the three dose groups, the 4, 4, 8 mg/kg dose did not consistently achieve the target range based on preclinical models. Further study of the relationship between ataluren concentration and activity is required. Nonetheless, the results of the in vitro myotube experiment (ie, increases in dystrophin expression in 100% [35/35] of samples) indicate that even non-responders by muscle biopsy in this study had the potential to respond at the cellular level.

This study highlights the challenges and limitations of currently available methods to assess changes in dystrophin expression. The value of muscle biopsy dystrophin expression as a biomarker in therapeutic trials targeting small increases in dystrophin levels has been questioned, because specimens only provide local information on muscle quality that may not reflect the state of all muscles. [15] Typically, a bilateral muscle is sampled from one side of the body before treatment and from the other side after treatment; thus, any differences between the left and right muscles introduce variability. Furthermore, a DMD muscle is often heterogeneous with respect to fibrofatty replacement of muscle, so that sampling error during biopsy may cause additional variability. We aimed to mitigate potential sampling-related variability by excising and evaluating the entire EDB muscle. The EDB muscle is distally located, little used, and therefore unlikely to demonstrate substantial fibrofatty replacement. Analysis of changes in dystrophin expression is hampered also by the lack of a sensitive, robust, and reproducible assay for quantifying low levels of dystrophin.

In this study, we initially performed a qualitative analysis of dystrophin expression. Subsequently, a quantitative analysis was applied to the same images that were examined qualitatively. Quantification of very low levels of dystrophin expression in immunostaining images of muscle samples presents numerous technical challenges. [16] Different methods (eg immunostaining and Western blot) applied to the same specimens result in inconsistent dystrophin levels. [17], [18] Western blotting analysis was not conducted in this study because there was insufficient biopsy material remaining, post-immunostaining, to perform Western blot analysis. Although the individual patient results were not always concordant and the overall results of positive changes in dystrophin expression amongst the two methods were not entirely consistent, both the qualitative and quantitative methods showed post-treatment increases in dystrophin in this study (Table 2). Collectively, these data support ataluren’s activity as a dystrophin restoration therapy in patients with nmDMD.

With regard to the other measure of pharmacodynamic activity in this study, serum CK reductions were observed in 84% (31/37) of subjects during ataluren administration. These changes were statistically significant at the 10, 10, 20 mg/kg and 20, 20, 40 mg/kg dose levels, but not at the 4, 4, 8 mg/kg dose level. However, using CK as a pharmacodynamic endpoint in DMD is also problematic. Although CK levels decreased in this study on treatment, changes in serum CK levels are highly variable and difficult to interpret. Decreases in serum CK levels may reflect a treatment effect or reduction in activity, while increases may indicate lack of a treatment effect or exercise-related stress to the body. [15], [19] Given the limitations in current quantitative dystrophin assays and CK, a validated biomarker for monitoring of drug activity in DMD is needed to facilitate drug development in this disease. Ultimately, clinically meaningful outcome measures such as the 6-minute walk test are required to evaluate therapeutic benefit in DMD [20], [21].

In this short-term study clinical improvements were not observed in timed function tests and myometry, which is not unexpected for a 28-day treatment with a drug intended to increase dystrophin production. Anecdotal reports of symptomatic effects at home and in the classroom were encouraging. Ataluren was generally well-tolerated in this study. Mild treatment-emergent adverse events of transient gastrointestinal complaints were observed at all three dose levels and appeared consistent with background symptoms commonly observed in clinical trials. No clearly dose-dependent increases in frequency or severity were evident. Episodes of nausea and vomiting were primarily related to anesthesia administered at the time of muscle biopsies. No drug-related serious adverse events were reported. No subject discontinued ataluren due to an adverse event.

Mean values for maximum concentration (C_max_) and AUC_0–24_ for ataluren plasma concentrations on Days 1 and 27 showed no difference in ataluren exposures in boys receiving or not receiving corticosteroids in DMD. At the 10, 10, 20 mg/kg and 20, 20, 40 mg/kg dose level, mean trough plasma concentrations achieved or exceeded target levels active in the *mdx* mouse model of DMD. Considerable intersubject variability was observed in ataluren exposure at all dose levels.

The presence of a nonsense codon in a transcript can affect the level of the mRNA, although the precise “rules” governing nonsense-mediated mRNA decay (NMD) have not been completely defined. In different cell lines harboring the CFTR W1282X mutation, NMD efficiency was shown to correlate with response to the stop codon readthrough compound gentamicin, as measured by restored chlorine channel activity. [22] Down regulating NMD by reducing UPF1 or UPF2 levels increased chloride channel activity in response to gentamicin in cell culture, consistent with the notion that transcript levels may act as a disease modifier. The applicability of modifying NMD for DMD has yet to be investigated.

The activity, safety, and pharmacokinetic findings from this initial study of ataluren in nonsense mutation DMD warranted advancement of this new drug candidate into larger, placebo-controlled testing. Given its potential to safely restore dystrophin production and thereby modify the course of this severely disabling disease affecting pediatric males, efforts to develop ataluren as a treatment for nonsense mutation DMD should remain an important priority for the DMD research community.

## Supporting Information

Figure S1 **In Vivo Dystrophin Expression by Immunofluorescence in Extensor Digitorum Brevis Muscle: Example of a Non-Responder Subject.** Subject received ataluren 10, 10, 20 for 28 days. Subject was judged visually as a non-responder and had a 22.1% decrease in dystrophin expression on the quantitative immunostain assay. **Abbreviation:** DMD = Duchenne muscular dystrophy.
(TIF)

Checklist S1 **CONSORT Checklist.**
(DOC)

Protocol S1 **Phase 2a Clinical Protocol.**
(DOCX)

Appendix S1 **Supplemental Appendix.**
(DOC)
