# Supplementary figures and images for: Phase 2a Study of Ataluren-Mediated Dystrophin Production in Patients with Nonsense Mutation Duchenne Muscular Dystrophy
Source: PLoS One. 2013 Dec 11;8(12):e81302. doi: 10.1371/journal.pone.0081302 (PMC3859499; doi:10.1371/journal.pone.0081302)

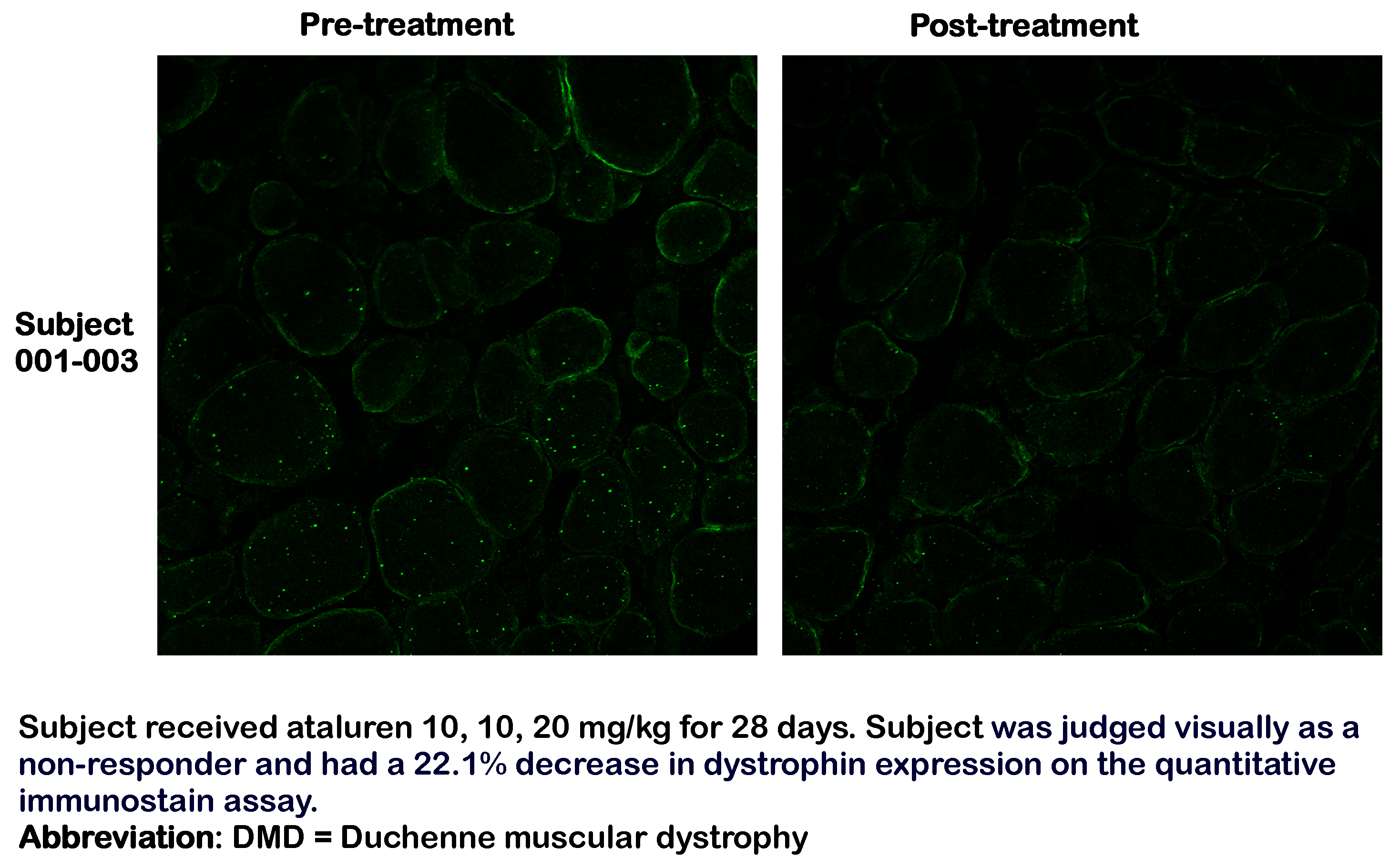

Supplement: Figure S1 — In Vivo Dystrophin Expression by Immunofluorescence in Extensor Digitorum Brevis Muscle: Example of a Non-Responder Subject. Subject received ataluren 10, 10, 20 for 28 days. Subject was judged visually as a non-responder and had a 22.1% decrease in dystrophin expression on the quantitative immunostain assay. Abbreviation: DMD = Duchenne muscular dystrophy. (TIF) [file pone.0081302.s001.tif]
