# Supplementary material for: Phase 2a Study of Ataluren-Mediated Dystrophin Production in Patients with Nonsense Mutation Duchenne Muscular Dystrophy
Source: PLoS One. 2013 Dec 11;8(12):e81302. doi: 10.1371/journal.pone.0081302 (PMC3859499; doi:10.1371/journal.pone.0081302)
Supplement: Protocol S1 — Phase 2a Clinical Protocol. (DOCX) [file pone.0081302.s003.docx]

CLINICAL PROTOCOL

A Phase 2 Study of PTC124 as an Oral Treatment for
Nonsense-Mutation-Mediated Duchenne Muscular Dystrophy

Protocol Number PTC124-GD-004-DMD

04 October 2006

PTC Therapeutics, Inc.

100 Corporate Court

South Plainfield, NJ 07080

PROTOCOL Identifiers

|  |  |
| --- | --- |
| **Therapeutic Area** | Genetic Disorders – Duchenne Muscular Dystrophy |
| **PTC Therapeutics Substance Identifier** | PTC124 |
| **IND Number** | 68,431 |
| **Included in clinical trials.gov Database** | Yes |
| **Protocol Number** | PTC124-GD-004-DMD |
| **Protocol Phase** | Phase 2 |
| **Protocol Title** | A Phase 2 Study of PTC124 as an Oral Treatment for Nonsense-Mutation-Mediated Duchenne Muscular Dystrophy |

TABLE OF CONTENTS

[1. Study Objectives and Endpoints 9](#_Toc358119068)

[1.1. Primary Objective 9](#_Toc358119069)

[1.2. Secondary Objectives 9](#_Toc358119070)

[1.3. Primary Endpoint 9](#_Toc358119071)

[1.4. Secondary Endpoints 9](#_Toc358119072)

[1.5. Rationale for Endpoints Selection 10](#_Toc358119073)

[2. Design and Design Rationale 12](#_Toc358119074)

[2.1. Design Overview 12](#_Toc358119075)

[2.2. Design Rationale 13](#_Toc358119076)

[3. Subject Selection 13](#_Toc358119077)

[3.1. Source and Number of Subjects 13](#_Toc358119078)

[3.2. Subject Selection Criteria 13](#_Toc358119079)

[3.2.1. Overview 13](#_Toc358119080)

[3.2.2. Inclusion Criteria 14](#_Toc358119081)

[3.2.3. Exclusion Criteria 15](#_Toc358119082)

[3.3. Enrollment Criteria Rationale 15](#_Toc358119083)

[4. Enrollment Procedures 16](#_Toc358119084)

[4.1. Registration 16](#_Toc358119085)

[4.2. Enrollment of Additional Subjects 17](#_Toc358119086)

[5. Study Drug administration 17](#_Toc358119087)

[5.1. Trial Product 17](#_Toc358119088)

[5.1.1. Description 17](#_Toc358119089)

[5.1.1.1. 4-, 4-, 8-mg/kg and 10-, 10-, 20-mg/kg doses 17](#_Toc358119090)

[5.1.1.2. 20-, 20, 40-mg/kg dose 18](#_Toc358119091)

[5.1.2. Preparation of Individual Doses and Dose Calculation 18](#_Toc358119092)

[5.1.2.1. 4-, 4-, 8-mg/kg and 10-, 10-, 20-mg/kg doses 18](#_Toc358119093)

[5.1.2.2. 20-, 20-, 40-mg/kg dose 19](#_Toc358119094)

[5.1.3. Drug Dispensing and Return of Medication 19](#_Toc358119095)

[5.1.3.1. 4-, 4-, 8-mg/kg and 10-, 10-, 20-mg/kg doses 19](#_Toc358119096)

[5.1.3.2. 20-, 20-, 40-mg/kg dose 20](#_Toc358119097)

[5.1.4. Storage and Stability 20](#_Toc358119098)

[5.1.4.1. 4-, 4-, 8-mg/kg and 10-, 10-, 20-mg/kg doses 20](#_Toc358119099)

[5.1.4.2. 20-, 20-, 40-mg/kg dose 20](#_Toc358119100)

[5.1.5. Source 20](#_Toc358119101)

[5.1.6. Overdose Precautions 21](#_Toc358119102)

[5.1.7. Inadvertent Exposure and Spill Precautions 21](#_Toc358119103)

[5.1.8. Study Medication Accountability 21](#_Toc358119104)

[5.2. Treatment 22](#_Toc358119105)

[5.3. Safety Evaluation and Treatment Modifications 23](#_Toc358119106)

[5.3.1. Safety Evaluation 23](#_Toc358119107)

[5.3.2. Treatment Modifications 24](#_Toc358119108)

[5.4. Diet 25](#_Toc358119109)

[5.5. Concomitant and Supportive Therapy 25](#_Toc358119110)

[5.6. Study Drug Administration Rationale 25](#_Toc358119111)

[6. Schedule of Events and study parameters 26](#_Toc358119112)

[6.1. Schedule of Events 26](#_Toc358119113)

[6.2. Explanation of Study Procedures 27](#_Toc358119114)

[6.2.1. Pretreatment, Treatment, and Follow-up Periods 27](#_Toc358119115)

[6.2.2. Dystrophin Gene Sequencing 28](#_Toc358119116)

[6.2.3. Hepatitis and HIV Screen 28](#_Toc358119117)

[6.2.4. Vital Signs 28](#_Toc358119118)

[6.2.5. Height, Weight, and Physical Examination 28](#_Toc358119119)

[6.2.6. Hematology Laboratory Assessment 28](#_Toc358119120)

[6.2.7. Biochemistry Laboratory Assessment 28](#_Toc358119121)

[6.2.8. Coagulation Laboratory Assessment 29](#_Toc358119122)

[6.2.9. Urinalysis 29](#_Toc358119123)

[6.2.10. 12-Lead ECG 29](#_Toc358119124)

[6.2.11. PTC124 Administration 29](#_Toc358119125)

[6.2.12. PTC124 Compliance 29](#_Toc358119126)

[6.2.13. Adverse Events 30](#_Toc358119127)

[6.2.14. Follow-up of Adverse Event or Abnormal Laboratory/ECG Findings 30](#_Toc358119128)

[6.2.15. Concomitant Medications 30](#_Toc358119129)

[6.2.16. Muscle and Skin Biopsies 31](#_Toc358119130)

[6.2.17. Upper and Lower Extremity Myometry 32](#_Toc358119131)

[6.2.18. Timed Function Tests 32](#_Toc358119132)

[6.2.19. Blood for Analysis of PTC124 Pharmacokinetics and Metabolism 32](#_Toc358119133)

[6.2.20. Long-term Follow-up 34](#_Toc358119134)

[6.3. Blood Collection Summary 34](#_Toc358119135)

[6.4. Study Parameter Selection Rationale 34](#_Toc358119136)

[7. Adverse Event Assessments 35](#_Toc358119137)

[7.1. Adverse Event Definitions 35](#_Toc358119138)

[7.1.1. Adverse Events 35](#_Toc358119139)

[7.1.2. Serious Adverse Events 36](#_Toc358119140)

[7.1.3. Unexpected Adverse Events 36](#_Toc358119141)

[7.2. Eliciting Adverse Event Information 37](#_Toc358119142)

[7.3. Adverse Event Recording 37](#_Toc358119143)

[7.4. Adverse Event Reporting Requirements 38](#_Toc358119144)

[7.5. Adverse Event Reporting Period 38](#_Toc358119145)

[7.6. Grading of Adverse Event Severity 39](#_Toc358119146)

[7.7. Describing Relationship to Study Drug 39](#_Toc358119147)

[7.8. Follow-up of Unresolved Adverse Events 39](#_Toc358119148)

[8. Withdrawal of subjects From Study Participation 40](#_Toc358119149)

[9. Statistics and Data Management 41](#_Toc358119150)

[9.1. Basis for Dose Escalation (4-, 4-, and 8-mg/kg to 10-, 10-, and 20-mg/kg) 41](#_Toc358119151)

[9.2. Basis for Dose Escalation (10-, 10-, and 20-mg/kg to 20-, 20-, and 40-mg/kg) 41](#_Toc358119152)

[9.3. Power Calculation Based on Proposed Sample Size at the 10-, 10-, and 20-mg/kg Dose Level 41](#_Toc358119153)

[9.4. Analysis Plan 42](#_Toc358119154)

[9.5. Data Management and Quality Assurance 44](#_Toc358119155)

[9.6. Data Monitoring Committee 44](#_Toc358119156)

[9.7. Statistical and Data Management Rationale 45](#_Toc358119157)

[10. Obligations of the Investigator and the Sponsor 45](#_Toc358119158)

[10.1. Compliance with Ethical and Regulatory Guidelines 45](#_Toc358119159)

[10.2. Institutional Review Board 45](#_Toc358119160)

[10.3. Informed Consent/Assent 46](#_Toc358119161)

[10.4. Electronic Case Report Forms 46](#_Toc358119162)

[10.5. Study Records 47](#_Toc358119163)

[10.6. Confidentiality 47](#_Toc358119164)

[10.7. Retention of Records 47](#_Toc358119165)

[10.8. Monitoring and Auditing 48](#_Toc358119166)

[10.9. Termination of the Study 48](#_Toc358119167)

[10.10. Dissemination of Results 49](#_Toc358119168)

[11. Bibliography 50](#_Toc358119169)

TABLES

[Table 1. Dosing Scheme 23](#_Toc358119057)

[Table 2. DMD Dose-Ranging Phase 2a Study Schedule of Events 27](#_Toc358119058)

[Table 3. Schedule of Pharmacokinetic Sample Collection 33](#_Toc358119059)

[Table 4. Blood Drawing Requirements 34](#_Toc358119060)

[Table 5. Preliminary PTC124 Safety Profile 37](#_Toc358119061)

[Table 6. Reporting Requirements For Adverse Events 38](#_Toc358119062)

[Table 7. Grading of Adverse Event Severity (Events not Graded in CTCAE) 39](#_Toc358119063)

[Table 8. Relationship of Study Drug To Adverse Event 39](#_Toc358119064)

[Table 9. Statistical Basis for Dose Escalation 41](#_Toc358119065)

[Table 10. Exact Binomial Confidence Intervals by Dystrophin Response Rate (10-, 10-, and 20-mg/kg dose only) 42](#_Toc358119066)

[Table 11. Exact Binomial Confidence Intervals by Dystrophin Response Rate 43](#_Toc358119067)

Abbreviations

| **Abbreviation** | **Definition** |
| --- | --- |
| A | Adenosine |
| ADME | Absorption, distribution, metabolism and excretion |
| ALT | Alanine aminotransferase |
| ANOVA | Analysis of variance |
| aPTT | Activated partial thromboplastin time |
| AST | Aspartate aminotransferase |
| AUC_0-12_ | Area under the concentration versus time curve from time 0 to 12 hours |
| AUC_0-24_ | Area under the concentration versus time curve from time 0 to 24 hours |
| BID | Bis in die (twice per day) |
| C | Cytidine |
| CF | Cystic fibrosis |
| C_max_ | Maximum plasma concentration |
| CFR | Code of Federal Regulations |
| CFTR | Cystic fibrosis transmembrane conductance regulator |
| cGMP | Current Good Manufacturing Practices |
| CHO | Chinese hamster ovary |
| CI | Confidence interval |
| CK | Creatine kinase |
| CRF | Case report form |
| CRO | Contract Research Organization |
| CTCAE | Common Terminology Criteria for Adverse Events |
| DMC | Data Monitoring Committee |
| DMD | Duchenne muscular dystrophy |
| DNA | Deoxyribonucleic acid |
| ECG | Electrocardiogram(s) |
| EDB | Extensor digitorum brevis |
| EDTA | Ethylenediaminetetraacetic acid |
| FBS | Fetal bovine serum |
| FDA | Food and Drug Administration |
| G | Guanosine |
| GAPDH | Glyceraldehyde-3-phosphate dehydrogenase |
| GCP | Good Clinical Practices |
| GGT | Gamma-glutamyl transferase |
| GLP | Good Laboratory Practices |
| HDPE | High density polyethylene |
| HEK | Human embryonic kidney (cells) |
| hERG | Human ether-à-go-go-related gene |
| HIV | Human immunodeficiency virus |
| HPLC-MS-MS | High performance liquid chromatography with tandem mass spectrometry |
| ICH | International Conference on Harmonisation |
| IND | Investigational New Drug (Application) |
| INR | International Normalized Ratio |
| IRB | Institutional Review Board |
| LDH | Lactate dehydrogenase |
| MedDRA | Medical Dictionary for Regulatory Activities |
| MRI | Magnetic resonance imaging |
| mRNA | Messenger ribonucleic acid |
| MTD | Maximum tolerated dose |
| NDA | New Drug Application |
| NOAEL | No-observed-adverse-effect-level |
| PBMCs | Peripheral blood mononuclear cells |
| PK | Pharmacokinetic(s) |
| PPARγ | Peroxisome proliferator-activated receptor gamma |
| PT | Prothrombin time |
| QT | Ventricular depolarization-repolarization interval on ECG |
| RCF | Relative centrifugal force |
| SAS | Statistical Analysis System |
| T | Thymidine |
| t_½_ | Terminal elimination half-life |
| TA | Tibialis anterior |
| TID | Ter in die (three times per day) |
| T_max_ | Time that C_max_ was observed |
| U | Uridine |
| UCP1 | Uncoupling protein 1 |

# Study Objectives and Endpoints

## Primary Objective

- To determine whether PTC124 safely provides pharmacological activity as measured by immunofluorescence evidence of an increase in dystrophin production on extensor digitorum brevis (EDB) or tibialis anterior (TA) muscle biopsy

## Secondary Objectives

- To evaluate the effect of PTC124 on additional markers of disease activity
- To assess changes in muscle function
- To evaluate PTC124 effects on dystrophin mRNA in EDB/TA muscle
- To determine compliance with PTC124 therapy
- To characterize the safety profile of PTC124 in subjects with DMD
- To determine the PK profile of PTC124 in subjects with DMD
- To explore PTC124 activity in in vitro myocyte and dermal fibroblast cultures derived from biopsy tissues

## Primary Endpoint

- Immunofluorescence evidence of a change in dystrophin expression on EDB/TA muscle biopsy, defined as an increase between the baseline biopsy and the end-of-treatment biopsy) in the staining of the sarcolemmal membrane with an antibody to the C-terminal portion of the dystrophin protein (excluding revertant fibers)

## Secondary Endpoints

- Immunofluorescence evidence of changes in sarcoglycans and dystroglycan in EDB/TA muscle biopsy specimens
- Western blot evidence of changes in dystrophin, sarcoglycans, and dystroglycan in EDB/TA muscle biopsy specimens
- Presence of dystrophin mRNA in EDB/TA muscle as determined by quantitative polymerase chain reaction (PCR) assay
- Changes in serum CK levels
- Quantitative muscle strength of upper and lower extremities as assessed by measurement of force at hip, knee, and elbow joints, and of hand grip through myometry testing
- Time taken to stand from supine position, time taken to walk 10 meters, and time taken to climb 4 standard-sized steps
- Study drug compliance as assessed by quantification of used and unused drug
- Safety profile characterized by type, frequency, severity, timing, and relationship to study drug of any adverse events, laboratory abnormalities, or ECG abnormalities
- PK parameters, eg, T_max_, C_max_, AUC, t_1/2_
- Dystrophin and dystrophin-associated protein expression by immunofluorescence and Western blotting in cultured myocytes and dermal fibroblasts in response to PTC124 treatment (exploratory)

## Rationale for Endpoints Selection

The proposed pharmacodynamic, efficacy, safety, and PK tests are chosen based on relevance to the pathophysiology and clinical manifestations of the disease, and on past experience that these tests can be performed with acceptable reliability. All of the endpoints have also been employed in prior trials of therapy for DMD. The following considerations have been taken into account in selecting endpoints for evaluation in the Phase 2a DMD clinical trial.

- **Dystrophin, sarcoglycan, and dystroglycan expression by immunofluorescence and Western blotting**: Muscle biopsies are commonly performed on DMD subjects as a component of diagnosis and as measures of therapeutic effect in the context of research studies. Immunofluorescence and Western blotting are routine tests performed on muscle biopsy specimens to confirm the presence or absence of full-length dystrophin. An absence or low-level staining of dystrophin in the muscle is viewed as confirmation of the diagnosis of DMD. Restoration of dystrophin or increase in the staining for dystrophin, with localization to the muscle membrane, has been considered a direct measure of preclinical and clinical pharmacodynamic activity in assessments of gentamicin as a potential treatment for nonsense mutation suppression [Barton-Davis 1999, Politano 2003]. Similarly, induction of dystrophin expression as measured by immunofluorescence and Western blotting in the *mdx* mouse model has documented PTC124 activity [Barton 2005].

Dystrophin-associated-proteins, such as various sarcoglycans and dystroglycan, form a complex at the muscle membrane that is critical in maintaining muscle integrity and physiologic function. An absence of dystrophin, as in DMD, results in a corresponding reduction of sarcoglycans and dystroglycan at the muscle membrane [Ohlendieck 1991]. Conversely, production of an adequate amount of functional dystrophin is associated with the reappearance of these proteins at the muscle membrane. Immunofluorescence and Western blotting evaluation of sarcoglycans and dystroglycan therefore offer confirmatory evidence of the presence of functional dystrophin. PTC124 treatment in the *mdx* mouse model has been shown to induce the appearance of sarcoglycans when assessed by Western blotting [Barton 2005].

In this clinical study, the EDB muscle will be biopsied from one foot for confirmation of the absence of dystrophin prior to treatment initiation and from the other foot to assess for production of dystrophin following PTC124 administration. A standard procedure for sampling of the EDB muscles from the feet has been established and has been used for assessing the presence of dystrophin and γ sarcoglycan [Stedman 2000]. EDB has been chosen because it is not an essential muscle for daily activities and therefore sampling this muscle does not have adverse functional consequence for the subject. Because it is little used, the EDB muscle is unlikely to demonstrate substantial fibrotic replacement of muscle and thus provides an appropriate tissue for detection of dystrophin production. Sampling of the EDB muscle offers additional practical advantages because it is easy to identify, can be dissected under local anesthesia, and provides sufficient amounts of tissue to carry out the required analyses. Considering that some patients with DMD undergo lower extremity surgery at an early age, EDB muscle may not be available. Thus, in such patients, the TA muscle will be biopsied using a standardized procedure.

- **Serum CK**: Serum CK provides a measure of whole-body muscle integrity. Concentrations of this enzyme in the serum are increased 50- to 100-fold in subjects with DMD and measurements of its levels are used in making an early diagnosis of the disease [Worton 2001]. The levels of serum CK are measured to monitor the progression of the disease and serve as a marker for muscle damage. While exercise-induced changes introduce variability [Politano 2003], the marker has advantages because it can be easily, repeatedly, and frequently assessed with a widely available and reliable assay. Prior clinical studies have shown decreases in serum CK coincident with improvements in muscle strength during treatment with steroids [Drachman 1974, Mendell 1989, Reitter 1995]. In preclinical studies in the *mdx* mouse, PTC124 treatment has induced significant decreases in serum CK values within 2 weeks of initiating treatment [Barton 2005].
- **Upper and lower extremity myometry**: Myometry assessments using a hand-held dynamometer offer a sensitive and reproducible measure of muscle strength in ambulatory and non-ambulatory subjects [Stuberg 1988, Brussock 1992, McDonald 1995, Beenakker 2001, Hyde 2001]. Inter-rater reliability in subjects with muscular dystrophy is high [Stuberg 1988, Hyde 2001] and normative values in children between the ages of 4 and 16 years have been established [Beenakker 2001]. As compared to manual muscle strength testing [Florence 1992], myometry is a more sensitive and less complex measure of muscle function [McDonald 1995]. The test is easy to perform and can be readily administered by the evaluator (eg, physician or physical therapist) in the neuromuscular clinics participating in the study.
- **Timed function tests**: These tests (time taken to stand from supine position, time taken to walk 10 meters, and time taken to climb 4 standard-sized steps) provide an additional measure of functional capability in ambulatory subjects. The tests are reproducible, commonly employed, simple to administer, and have documented response to therapeutic intervention with steroids [Mendell 1989, Griggs 1991].
- **Study drug compliance**: Evaluation of study drug compliance provides context for assessments of pharmacologic activity, safety, and PK, and may offer a general indication of subject acceptance of therapy, integrating factors of tolerability, palatability, and convenience. Subjects will be asked to record the date and time of day (ie., breakfast, lunch, or dinner) at which the drug was taken and will be questioned for the reason for any missed doses (see Study Manual). The compliance of the subject will be verified by counting used and unused drug bottles. Clinic staff will record the study medication dosing information, including the exact clock time of each dose, during the time the subject is confined to the clinic.
- **Safety**: Safety will be characterized in terms of the type, incidence, timing, severity, and relatedness of adverse events to study drug, individually for each subject. For consistency of interpretation, adverse events will be coded using the standard Medical Dictionary for Regulatory Activities (MedDRA) (see Study Manual) and the severity of these events will be graded using the well-defined Common Terminology Criteria for Adverse Events (CTCAE) Version 3.0 (see Study Manual).
- **Pharmacokinetics**: Given the importance of characterizing the PK of PTC124 in children, of establishing exposure-response relationships, and of providing necessary information for product labeling, all subjects enrolled to the study will participate in the PK sampling component of this study. Sampling on Day 1 and Day 27 after each of the 3 doses on those days will provide information about potential changes in exposure over time and possible diurnal variation in plasma concentration-time curves. The high-performance liquid chromatography-tandem mass spectroscopy (HPLC-MS-MS) method used to quantify PTC124 plasma concentrations has been fully validated in the context of the prior Phase 1 studies.
- **Dermal fibroblast and muscle cell culture**: Companion laboratory studies will be performed on muscle tissue and skin from subjects participating in the study. These exploratory investigations seek to determine whether PTC124-induced dystrophin production in primary muscle cultures from the subjects will correspond with PTC124-induced dystrophin production in vivo. In addition, these experiments will evaluate whether dermal fibroblasts from subjects, when differentiated into muscle cells in vitro by transfection with a Myo-D-producing expression construct [Wang 2001], will demonstrate dystrophin production in response to PTC124 treatment. Correlations of skin cell response with clinical activity may offer an easy-to-obtain predictive test in selecting future subjects for PTC124 therapy or for screening new agents for the treatment of DMD.

# Design and Design Rationale

## Design Overview

This is a Phase 2a, multi-site, open-label, dose-ranging, efficacy, safety, and PK study of PTC124 in approximately 32 subjects with nonsense-mutation-mediated DMD. It is anticipated that 3 centers in the US will participate. In the first part of the study, 6 subjects will receive PTC124 at a low dose of 4-, 4- and 8-mg/kg (given TID for 28 days). Once these subjects have completed 28 days of treatment, ~18 additional subjects will be enrolled and will receive PTC124 at a middle dose of 10-,10- and 20-mg/kg (given TID for 28 days). Following review of the preliminary safety and PK data from the patients who have completed treatment at the low- or mid‑dose, 6 to12 additional subjects will be enrolled to receive 28 days of treatment with PTC124, given TID at a high dose of 20‑, 20-, and 40-mg/kg, to further evaluate the drug’s safety, PK profile, and pharmacological activity. The study will be conducted through academic centers that are experienced in the conduct of clinical trials involving subjects with muscular dystrophy. Enrollment, follow-up, and analysis are planned to occur over approximately 24 months for the treatment and initial follow-up portions of the study. Long-term follow-up for 5 years, as measured from the start of study treatment, will be subsequently be reported (see Section 6.2.20).

The proposed trial will be performed in accordance with Good Clinical Practice (GCP) guidelines. Subjects will be monitored closely and results will be subject to review by regulatory authorities and an Institutional Review Board (IRB). A subject (and/or his parent/legal guardian) must provide written informed consent prior to study drug administration. If the subject is < 18 years of age but ≥ 7 years of age, he must also provide a written assent, unless the age for assent differs according to local IRB requirements. The age for obtaining assent as defined by the responsible IRB should be followed.

## Design Rationale

The proposed Phase 2a study has been designed to build on the Phase 1 experience with PTC124, and to safely and efficiently generate critical dosing, activity, toxicity, and PK data in support of future clinical development of PTC124. Inclusion of multiple sites enhances study accrual potential from this limited subject population while providing reassurance that the results are likely to have general applicability. The open-label design is considered acceptable in the initial screening of a new therapeutic agent, and baseline evaluations allow comparison of study parameters on and off PTC124 treatment. The design also allows efficient collection of
short-term activity and safety data in the limited numbers of subjects with nonsense-mutation-mediated DMD. Because plasma exposures were lower than anticipated in patients with DMD at the low and middle dose levels, further dose escalation to a dose level of 20-, 20-, and 40-mg/kg will allow safety and PK assessments at target plasma concentrations known to be maximally active in preclinical animal models and shown to be safe in healthy volunteers and patients with CF. Conduct through academic centers provides the assurance that the site will have a dedicated DMD clinic, a general clinical research ward, an onsite pharmacy, a clinical laboratory for analysis of safety parameters and PK sample processing, and facilities for assessment of other parameters required within the context of this protocol. Centralized analysis of efficacy data from specialized studies such as immunofluorescence of muscle biopsies will optimize consistency of analyses across subjects and, therefore, interpretability of results.

A total accrual, treatment, follow-up, and analysis time of approximately 24 months for the treatment and initial follow-up portions of the study is anticipated based on the past experience of the investigators who will be involved in the study. Because PTC124 is a new chemical entity with a novel mechanism of action, patients receiving the drug will undergo long-term follow-up in order to build a safety database regarding the potential for any late toxicities. Implementation of the study under GCP maximizes subject safety and helps ensure conformance with regulations in the context of a registration-directed program.

# Subject Selection

## Source and Number of Subjects

Subjects will be enrolled from the participating academic centers and will undergo study-specific screening procedures prior to enrollment. The goal is to have 32 subjects enrolled to receive PTC124 at one of 3 dose levels for the intended 28 days of treatment. A maximum total enrollment of 36 subjects is anticipated to ensure that 32 subjects complete the study treatment.

## Subject Selection Criteria

### Overview

This clinical trial can fulfill its objectives only if appropriate subjects are enrolled. The following eligibility criteria are designed to select subjects for whom study participation is considered appropriate. All relevant medical and non-medical conditions should be taken into consideration when deciding whether this protocol is suitable for a particular subject. Eligibility criteria may not be waived by the investigator and are subject to review in the case of a GCP or a regulatory authority audit. Any questions regarding a subject’s eligibility should be discussed with the PTC Therapeutics medical monitor (see protocol cover page) prior to enrollment.

### Inclusion Criteria

Subjects must meet all of the following conditions to be eligible for enrollment into the study:

1. Diagnosis of DMD based on a clinical phenotype presenting by age 5, with increased serum CK and diminished staining for dystrophin on a muscle biopsy (diminished sarcolemmal staining with an antibody to the C-terminal portion of the dystrophin protein). ***Note: For a subject who has not had a prior muscle biopsy and meets all other eligibility criteria, a biopsy from a muscle other than the EDB may be performed to establish eligibility or the baseline EDB muscle biopsy may be used for assessing eligibility.***
2. Presence of a nonsense mutation in the dystrophin gene.
3. Documentation that dystrophin gene sequencing has been performed by the University of Utah, Salt Lake City, UT or, if sequencing has not already been performed by the University of Utah, that a blood sample has been sent for the confirmatory dystrophin gene sequencing. ***Note: A subject who has already had gene sequencing performed at the University of Utah does not need to have a repeat test performed. A subject who has documentation of a nonsense mutation by other methods need not wait for confirmatory results to start study therapy as long as a blood sample has been sent to the University of Utah.***
4. Physical examination or radiographic imaging evidence of EDB muscles in both feet. ***Note: A subject who does not have physically palpable EDB muscles should undergo an ultrasound/magnetic resonance imaging (MRI) of the feet for documentation of the bilateral presence of EDB muscles that have not undergone fibrosis.*** ***Note: In a patient who does not have the EDB muscle available for biopsy, the TA muscle will be biopsied using a standardized procedure.***
5. Ability to ambulate or, if non-ambulatory, then not requiring ventilator support.
6. Male sex.
7. Age ≥5 years.
8. Willingness to abstain from sexual intercourse or employ a barrier or medical method of contraception during the study drug administration and follow-up periods in subjects known to be sexually active.
9. Willingness and ability to comply with scheduled visits, drug administration plan, laboratory tests, study restrictions, study procedures (muscle biopsies and PK sampling), and functional and myometry testing (for patients who are able to perform such testing).
10. Able to provide written informed consent if ≥18 years of age, or written informed assent (with parental/guardian consent) if ≥7 years of age. If the subject is <7 years of age, parent/legal guardian consent alone will be obtained. ***Note: As the age for assent may differ according to local IRB requirements, the age for obtaining assent as defined by the IRB should be followed.***
11. Evidence of personally signed and dated informed consent document (assent also required for children ≥7 years of age) indicating that the subject/parent/legal guardian has been informed of all pertinent aspects of the trial. ***Note: As the age for assent may differ according to local IRB requirements, the age for obtaining assent as defined by the IRB should be followed.***

### Exclusion Criteria

The presence of any of the following conditions will exclude a subject from study enrollment:

1. Prior or ongoing medical condition (eg, concomitant illness, psychiatric condition, alcoholism, drug abuse), medical history, physical findings, ECG findings, or laboratory abnormality that, in the investigator’s opinion, could adversely affect the safety of the subject, makes it unlikely that the course of treatment or follow-up would be completed, or could impair the assessment of study results.
2. Clinical symptoms and signs of congestive cardiac failure (American College of Cardiology/American Heart Association Stage C or Stage D) [Hunt 2001].
3. Positive hepatitis B surface antigen, hepatitis C antibody test, or human immunodeficiency virus (HIV) test.
4. Hemoglobin <10 g/dL.
5. Serum albumin <2.5 g/dL.
6. Abnormal GGT or total bilirubin (>laboratory’s upper limit of normal).
7. Abnormal renal function (serum creatinine >1.5 times laboratory’s upper limit of normal).
8. History of solid organ or hematological transplantation.
9. Ongoing immunosuppressive therapy (other than corticosteroids).
10. Exposure to another investigational drug within 28 days prior to start of study treatment.
11. Ongoing participation in any other therapeutic clinical trial.
12. Ongoing use of thiazolidinedione peroxisome proliferator-activated receptor gamma
    (PPAR γ) agonists, eg, rosiglitazone (Avandia® or equivalent) or pioglitazone (Actos® or equivalent)
13. Change in systemic corticosteroid therapy (eg, initiation of treatment; cessation of treatment; change in dose, schedule, or type of steroid) within 3 months prior to start of study treatment.
14. Treatment with systemic aminoglycoside antibiotics within 4 weeks prior to start of study treatment. ***Note: A subject may receive systemic antibiotics as clinically necessary for life-threatening infections during the study, although, if medically appropriate, an attempt should be made to avoid systemic aminoglycoside antibiotics.***

## Enrollment Criteria Rationale

The subject eligibility criteria are designed to limit enrollment to subjects who clearly have DMD based on clinical, laboratory, and genetic findings but are sufficiently well (both in terms of DMD and in terms of concomitant illness) to safely participate in study procedures and provide interpretable results. These eligibility criteria are generally consistent with those used in prior Phase 2 evaluations of nonsense mutation suppression with gentamicin [Wagner 2001, Politano 2003].

Considering the qualitative nature of dystrophin immunohistochemistry and the knowledge that some patients with premature stop mutations do demonstrate a low-level of dystrophin expression in the muscle membrane, the criterion that subjects have a negative or low-level of staining for dystrophin prior to study enrollment enhances the certainty that a positive post-treatment result is meaningful. Restriction of enrollment to subjects with a nonsense mutation as the basis for DMD avoids the therapy of subjects who have little chance of benefit; it is known from prior studies with gentamicin that full-length protein production was seen only in subjects harboring a nonsense mutation as the exclusive basis for disease [Wilschanski 2003]. Enrolling only subjects with bilaterally detectable EDB/TA muscles enhances the chances that adequate amounts of tissue will be available to perform all of the biopsy-related analyses.

Study of children is important given the early onset of the disease in childhood and the need to intervene when the disorder may still be tractable to therapy. After evaluation of the safety profile of the drug at the first 2 dose levels in the study, enrollment of non-ambulatory patients is considered appropriate as this will provide further information on the PK profile in adolescent patients with DMD and also will increase the understanding of the safety profile of the drug in this patient population.

While not a risk for most subjects likely to be enrolled to this study, restriction on eligibility relating to reproductive potential in any subjects known to be sexually active is important because PTC124 is a new chemical entity and its effects on fertility have not been fully characterized.

Minimal transaminase elevations (ALT, AST) have been observed in healthy volunteers receiving PTC124. As a consequence, subjects with overt liver dysfunction are excluded from this study as are subjects who have active/chronic hepatitis infections. In addition, subjects who have abnormalities of GGT and bilirubin are excluded. It is not practical to exclude subjects with DMD on the basis of AST or ALT elevations since these parameters are commonly elevated due to muscle breakdown in this disease setting.

While conventional supportive therapies will be permitted, efforts will be made to avoid use of concomitant medications (ie, aminoglycosides) that might confound interpretation of study results. In particular, if medically appropriate, investigators may substitute other antibiotics for aminoglycosides in subjects who require treatment for infections. Given that corticosteroids are commonly used in the treatment of DMD and are unlikely to confound the assessment of the primary outcome measure, enrollment of subjects receiving corticosteroids is permitted. Since alteration in serum CK levels or muscle function tests are known to occur over 1 to 2 months following changes in corticosteroid administration [Mendell 1989], it is mandatory that the doses and schedule of these medications be kept constant for ≥3 months prior to entry into the study in order to avoid misinterpretation of these study endpoints.

# Enrollment Procedures

## Registration

The investigator must inform each prospective subject and/or parent/legal guardian of the nature of the study, explain the potential risks, and obtain written informed consent/assent prior to performing any study-related screening procedures. Once the informed consent/assent has been signed, screening procedures for determination of eligibility can be initiated.

Once screening procedures have been completed, the site coordinator will send a completed Registration Form (see Study Manual) to PTC Therapeutics with the following information:

- Center number
- Subject number
- Subject date of birth
- Subject weight (in kilograms)
- Copy of the prior muscle biopsy report documenting absence of dystrophin (with subject identifiers obscured)
- Starting dose and number of bottles to be given
- Subject eligibility check list

In order for the subject to begin study treatment, PTC Therapeutics personnel will provide confirmation of eligibility by either fax or e-mail to the site and will assign the patient a unique patient enrollment number (3-digit site number followed by a 3-digit patient number). Any questions regarding the eligibility of a subject should be discussed with the PTC Therapeutics medical monitor.

## Enrollment of Additional Subjects

Enrollment of 36 subjects is permitted in order to ensure that 32 subjects complete the study treatment.

Should a subject withdraw prematurely during the study, an additional subject may be enrolled if, based on a review of the available safety data, the investigator and the PTC Therapeutics medical monitor agree that the events leading to the subject withdrawal are unlikely to constitute a safety risk for further subject enrollment. This additional subject will be enrolled at the same dose level as that being administered to the subject who withdrew, and will have a unique subject number.

# Study Drug administration

## Trial Product

### Description

#### 4-, 4-, 8-mg/kg and 10-, 10-, 20-mg/kg doses

PTC124 is provided as a vanilla-flavored, white to off-white powder for oral suspension. The drug is manufactured and formulated under current cGMP conditions. The powder is packaged in 40-mL plastic (high-density polyethylene [HDPE]) bottles sealed with foil seals and white plastic, childproof caps. Each bottle will contain 1000 mg of the drug substance. Inactive ingredients include refined polydextrose, mannitol, polyethylene glycol 3350, poloxamer 407, crospovidone, hydroxyethyl cellulose, vanilla flavor, magnesium stearate (non-bovine), and colloidal silica. Bottle labels will indicate the name and dose strength of the drug, the lot number, and the storage conditions (ie, refrigeration at 2° to 8°C). Space will be allocated on the label for the pharmacist or other clinical research staff to enter information regarding the patient number. Space will also be allocated for the subject to enter the date and mark the time of day (ie, breakfast, lunch, or dinner) when the drug is used.

#### 20-, 20, 40-mg/kg dose

PTC124 is provided as a vanilla-flavored, white to off-white powder for suspension. The drug is manufactured and formulated under cGMP conditions. As more fully described in the Investigator Brochure, the formulation includes binding and suspending agents, surfactants, and various minor excipients that aid in the manufacturing process. The powder is packaged in aluminum foil child-resistant sachets. Each sachet will contain 125, 250 or 1000 mg of the drug substance, which is 25.0% of the total formulation weight. Sachet labels indicate the identity and dose strength of the enclosed material (PTC124), the lot number, the “Use by” date, the storage conditions (ie, room temperature between 15-30°C), the necessary space to enter the patient and cycle numbers, and the applicable cautionary and regulatory statements.

### Preparation of Individual Doses and Dose Calculation

#### 4-, 4-, 8-mg/kg and 10-, 10-, 20-mg/kg doses

The dosing volume of study drug in milliliters will be based on milligrams of drug per kilogram of subject body weight. To assign the correct volume of suspension to be delivered to the subject, refer to the appropriate dosing table in Appendix B that provides the volume in milliliters corresponding to the dose to be administered for a given body weight in kilograms.

To suspend PTC124 for administration, the dosing spoon provided with the study medication should be used to add 17 mL of still, plain water directly to each 1000 mg bottle (resulting in a final concentration of 50 mg/mL in a 20-mL total volume of suspension). The handle of the dosing spoon is hollow and is calibrated to the milliliter for ease of measurement. Immediately after water is added, the bottle should be capped and shaken gently by hand for at least 30 seconds to achieve a homogeneous suspension. Although the reconstituted suspension may remain in the original plastic bottle for up to 24 hours before ingestion, it is recommended that the drug be taken shortly after reconstitution. If there is a delay of more than 15 minutes between reconstitution and dosing, the bottle should be reshaken gently by hand for at least 30 seconds.

For subjects requiring 1 bottle (≤20 mL) of PTC124 for an entire day of dosing, all 3 doses (breakfast, lunch, and dinner) are to be taken from the same bottle (1 bottle per **day**). For subjects requiring ≥20 mL but ≤40 mL of total volume per day, each dose (breakfast, lunch, and dinner) will be taken from a different bottle (1 bottle per **dose**). For subjects requiring ≥40 mL of total volume per day, the first 2 doses of the day (breakfast and lunch) will be taken from a different bottle (1 bottle per **dose**), and the last dose of the day (dinner) will be taken from 2 additional bottles (2 bottles per **dose**). The dinner dose will comprise one entire bottle plus a fraction of the second bottle. Refer to the appropriate dosing table in Appendix B and to the Study Manual for further details.

For all doses of PTC124, the desired volume should be withdrawn from the study medication bottle into the dosing spoon provided with the study medication bottle and dosed using the same spoon. After ingestion of study medication, the bottle(s) and/or dosing spoon should be filled with the same volume of tap water as the dose volume, and the water should be ingested. This rinse procedure should be carried out once. With the ingestion of the study medication and the rinse, study medication dosing will be considered complete.

During the 24 hours after reconstitution, >1 dose may be taken from a bottle of suspension; however, reconstituted study medication should not be stored beyond 24 hours with the intention of using this material again for multiple doses in the same subject.

Clinic staff will administer the study medication during the time the subject is confined to the clinic. The clinic staff will instruct the subject/parent/caregiver in the proper method for reconstituting and dosing the drug at home. In addition, detailed written drug reconstitution and dosing instructions will be provided to the subject/parent/caregiver when drug supplies are dispensed (see Study Manual). If English is not well understood by the person responsible for reconstitution and dosing, instructions will be provided in another appropriate language (eg, Spanish). The clinic staff will follow-up with the subject/parent/caregiver regarding the drug preparation and dosing procedures within 7 days after initiation of drug treatment.

#### 20-, 20-, 40-mg/kg dose

Dosing of study drug will be based on milligrams of drug per kilogram of patient body weight and in general, will be rounded to be consistent with the available sachet dose strength. The number of sachets to be taken for each dose with the 20-, 20-, and 40-mg/kg TID dose regimen is listed (by body weight) in Appendix B Table 4.

Study drug sachets should be stored at room temperature away from the reach of children until time of reconstitution. The suspension (powder in the sachet) may be reconstituted with still, plain water or apple juice or milk (skim, 1% fat, 2% fat, whole milk, or lactose-free milk). The number of sachets to be taken for a dose (as shown in Appendix B Table 4) should be separated from the total number of sachets dispensed by the pharmacy for the patient. All sachets to be taken for a dose should be cut open with scissors and the powder emptied out in a container (eg, a glass or cup) made out of plastic or glass. Approximately 30 to 50 mL of water, apple juice, or milk is then added to the powder in the container and the mixture is stirred with a spoon for ≥ 15 seconds before the patient drinks it. Additional water, apple juice, or milk is then added to the container to suspend any drug that might be sticking to the side or the bottom of the container and this rinse is also ingested to ensure that no powder is left in the container and to complete dosing.

### Drug Dispensing and Return of Medication

#### 4-, 4-, 8-mg/kg and 10-, 10-, 20-mg/kg doses

The clinic pharmacist or other qualified person will be responsible for dispensing study medication. Any unused study medication as well as partial and empty bottles will be returned to the clinic for inventory. The total amount of drug necessary for the entire 4-week period will be dispensed to the subject/parent/caregiver at the beginning of the treatment period. The clinic pharmacist or other qualified person will provide the subject/parent/caregiver with the specific dosing volume for that subject and with detailed directions regarding drug preparation.

Subjects should return all the remaining medication (any drug remaining in the opened bottles and the sealed bottles that were never opened) to the study site at the end of the treatment period. The Medication Administration Record will serve as the source document for drug supply to the subjects and will document the return of any unused drug for compliance assessments. Clinic staff will record the study medication administration information, including the exact clock time of each dose, during the time the subject is confined to the clinic.

#### 20-, 20-, 40-mg/kg dose

The clinic pharmacist or an alternative qualified person will be responsible for dispensing study medication. All used or unused study medication sachets will be returned to the clinic for inventory. The total amount of drug necessary for the entire 4-week dosing period will be dispensed to the patient/guardian at the beginning of the cycle. The clinic pharmacist or an alternative qualified person will provide each patient/guardian with the specific number of sachets to be taken at each dose and with detailed directions regarding drug preparation.

Subjects should return all the used sachets and the remaining medication (any sachets that were never opened) to the study site at the end of each treatment period. The Medication Administration Record will serve as the source document for study drug supplied to the patients and will document the return of used sachets and any unused drug for compliance assessments. Clinic staff will record the study medication administration information, including the exact clock time of each dose, during the time the subject is confined to the clinic.

### Storage and Stability

#### 4-, 4-, 8-mg/kg and 10-, 10-, 20-mg/kg doses

PTC124 study drug bottles must be shipped and stored under refrigerated conditions (2^o^ to 8^o^C). The patients should also be instructed to keep the drug under refrigerated conditions at home. However, unopened study drug bottles can be kept at room temperature for up to 48 hours. No specific warming of study drug is necessary before reconstitution. High-humidity storage conditions should be avoided as much as possible.

Stability data supports storage of the drug product in unopened bottles at 2^o^ to 8^o^C for at least 6 months. The clinical sites will be updated as more stability data become available.

The suspension must be reconstituted with still, plain water. Stability of PTC124 in liquids other than water has not been established. The reconstituted suspension with water has proved to be chemically and physically stable for at least 24 hours at room temperature under normal lighting conditions.

#### 20-, 20-, 40-mg/kg dose

Sachets containing PTC124 powder for oral suspension will be shipped to the site under appropriate conditions, and should be stored at room temperature (15^o^ to 30^o^C) at the site and by the patient at home. During shipping and storage, excursions in temperature outside of the range of 5^o^ to 40^o^C should be avoided.

Current stability data supports the use of PTC124 in this study. Clinical sites will periodically be updated as more stability data becomes available and the revised “Use by” date will be provided accordingly.

PTC124 suspended in water, apple juice, or milk (skim, 1% fat, 2% fat, whole milk, or lactose-free milk) is chemically and physically stable for at least 24 hours at room temperature under normal lighting conditions. Stability of PTC124 in liquids other than water, apple juice or milk has not been established.

### Source

PTC124 will be supplied free of charge to the clinical site by PTC Therapeutics for appropriate distribution to the subjects/caregivers. It is intended that the study drug will be provided in sufficient supply for the entire study. However, if necessary, resupply may be obtained by contacting the PTC Therapeutics project manager (see Study Manual).

### Overdose Precautions

In the Phase 1 single-dose study, the maximum tolerated dose was established to be 100 mg/kg. Doses of 150 and 200 mg/kg were associated with transient nausea, vomiting, diarrhea, headache, and/or dizziness; one subject receiving 200 mg/kg had reversible increases in AST and ALT (>2 times the upper limit of normal). Thus, administration of >100 mg/kg/dose of PTC124 should be considered an overdose.

For any patient experiencing overdose, observation and management for any symptomatic side effects (eg, nausea, vomiting, diarrhea, headache, or dizziness) should be instituted and biochemical and hematological parameters should be followed closely (consistent with the protocol [see Section 6] or more frequently, as needed). If the amount of drug ingested is
≥200 mg/kg/dose, gastric lavage or induction of emesis within 4 hours of drug ingestion may reduce exposure. The subsequent dose of PTC124 should be omitted; thereafter study drug administration may continue, if medically appropriate.

The PTC Therapeutics medical monitor should be contacted if an overdose (administration of >100 mg/kg/dose of PTC124) occurs. Under applicable regulations, overdosing is considered a serious adverse event and should be reported accordingly (see Sections 7.1.2 and 7.4).

### Inadvertent Exposure and Spill Precautions

Based on available data from nonclinical and clinical studies, PTC124 does not appear to be irritative and the drug is not acutely toxic at doses that are likely to result from inadvertent exposure. However, clinical personnel and caregivers should use reasonable precautions to avoid eye contact, skin contact, inhalation, or ingestion of the drug. In case of skin contact, the affected area should be washed thoroughly with water. In case of eye contact, rinse eyes thoroughly with water. If inhaled, remove to fresh air. If swallowed, rinse out mouth with water. If clinical symptoms such as headache, nausea, abdominal discomfort, or diarrhea are reported then administer supportive treatment for the symptoms and seek medical advice as soon as possible. The PTC Therapeutics medical monitor should also be called if inadvertent exposure occurs

In case of an accidental spill, protective (waterproof) gloves and eye protection should be worn during clean-up, if it is feasible to do so. If study medication powder or suspension is spilled, use disposable absorbent materials (eg, paper towels or sponge) to contain and collect the material into an airtight, waterproof, sealable container for conventional disposal. In either case, rinse the area of spillage thoroughly with water and dispose of the rinse liquid via the sewage system.

### Study Medication Accountability

Study personnel must ensure that all study medication supplies are kept in a secure locked area with access limited to authorized personnel. This study product must not be used outside the context of this protocol. Under no circumstances should the investigator or site personnel supply study product to other investigators or clinics, or allow the supplies to be used other than as directed by this protocol.

The investigator must maintain accurate records of the receipt of all study medication shipped by PTC Therapeutics or its designee, including, but not limited to, the date received, lot number, amount received, and the disposition of all study medication. Current dispensing records must also be maintained that include the date and amount of medication dispensed, relevant batch or bottle code numbers, and the subject’s assigned study number.

At the time of dispensing the drug to the subject, the drug will be identified by the subject’s enrollment number. A drug-dispensing log will be maintained at each site. At each clinic visit by the subject, including the end of the study visit, all empty or partially used bottles in their original cartons (4-, 4-, and 8mg/kg and 10-, 10-, and 20-mg/kg dose levels only), all used sachets and unused medication sachets (20-, 20-, and 40-mg/kg dose level only) and all unused medication must be returned to the site for accounting.

All used containers or sachets of study medication should be discarded according to standard institutional policy only after the study monitor has examined them. Depending upon the decision of PTC Therapeutics, unused clinical supplies may be destroyed on site as dictated by the appropriate standard procedures at the participating institution or must be returned to PTC Therapeutics or its designee after the study is completed. Records documenting the date of study medication destruction or shipping, relevant batch or bottle/sachet code numbers, and amount destroyed or shipped should be kept.

## Treatment

Treatment will be administered over 28 days for each treatment cohort. As shown in Table 1 below, 6 subjects will be treated daily for 28 days with PTC124 given at the low dose level
(ie, 4-, 4-, and 8-mg/kg) TID. If the initial 6 subjects tolerate the drug, then a second cohort of 18 subjects will receive PTC124 at the middle dose level (ie, 10-, 10-, and 20-mg/kg) TID. Upon the completion of the middle dose group, an additional 6 to 12 subjects will be enrolled to receive 28 days of treatment with PTC124, given TID at a dose of 20-, 20-, and 40-mg/kg. Thus, each subject will receive a total of 84 doses of PTC124. After the end of 28 days of treatment, each subject will be followed for an additional 28 days without study drug.

At each dose level, PTC124 should be taken TID at 6-, 6-, and 12-hour (± ~30 minutes) intervals. Ideally each dose should be taken within ~30 minutes after a meal (eg, ~7:00 AM after breakfast, ~1:00 PM after lunch, and ~7:00 PM after dinner) While it is realized that variations in dosing schedule may occur in the outpatient setting, the prescribed regimen (including dosing intervals and the relationship of dosing to meals) should be followed closely on the days of PK sample collection.

| Table 1. Dosing Scheme | | | |
| --- | --- | --- | --- |
|  | **Low Dose (n=6)** | **Middle Dose (n=20)** | **High Dose (n=6 to 12)** |
| **Regimen** | TID dosing  with food | TID dosing  with food | TID dosing  with food |
| **Schedule** | 28 days treatment 28 days follow-up | 28 days treatment 28 days follow-up | 28 days treatment 28 days follow-up |
| **Time** | **Dose** | | |
| **~7:00 AM** | 4 mg/kg | 10 mg/kg | 20 mg/kg |
| **~1:00 PM** | 4 mg/kg | 10 mg/kg | 20 mg/kg |
| **~7:00 PM** | 8 mg/kg | 20 mg/kg | 40 mg/kg |
| **Abbreviations**: TID = three times per day | | | |
|  | | | |

Subjects/caregivers will be instructed to make sure that the preparation and ingestion of the study medication is performed consistent with the information provided in Section 5.1.2.

For a subject who inadvertently has a delay in administration of study drug of <4 hours, the planned dose should be taken. For a subject who inadvertently has a delay in administration of study drug of ≥4 hours, the dose should not be taken. Study drug administration may continue but the missed dose should not be made up and the planned timing of subsequent study drug dosing should not be altered. A subject who vomits after ingestion of the drug should be instructed to contact the clinic staff in order to notify them of such an event; study drug administration may continue, if medically appropriate, but the vomited dose should not be made up and the planned timing of subsequent study drug dosing should not be altered.

Clinic staff will administer the study medication during the time the subject is confined to the clinic.

## Safety Evaluation and Treatment Modifications

### Safety Evaluation

All decisions concerning safety will be made by the investigator and the PTC Therapeutics medical monitor with the intention of maintaining the well-being of each subject participating in the study. Subjects will be closely monitored with clinical observations and safety laboratory testing (see Section 6 for details of study procedures). The available safety, PK, muscle biopsy, serum CK, muscle function, and study conduct information will be reviewed on an ongoing basis via regularly scheduled teleconferences (eg, at 2-week intervals) among the investigators and the PTC Therapeutics medical monitor. Meeting minutes will be generated at each meeting and included in the sponsor’s study files. Formal reports will not be prepared prior to or following these meetings.

It is understood that safety is a medical judgment that cannot be prospectively defined in detail. However, as general guidance, a subject will be considered to have tolerated a dose if the subject experiences no clinically significant drug-related adverse event or laboratory abnormality. Conversely, a subject will not be considered to have tolerated the dose if he experiences a clinically significant drug-related adverse event or laboratory abnormality during the study drug administration or post-administration follow-up period. The CTCAE, Version 3.0, will be used to grade the severity of the adverse events and laboratory abnormalities. Grade 3 or Grade 4 adverse events or laboratory abnormalities will be considered clinically significant, although recurrent or persistent Grade 2 events may also be considered clinically significant in certain circumstances.

### Treatment Modifications

After 6 subjects have completed the 28 days of treatment at the low dose level, the safety data for these subjects will be reviewed by the investigator(s) in coordination with the PTC Therapeutics medical monitor to determine whether to start enrollment of the middle dose level. Progression of the study to the middle dose level can occur once all subjects enrolled at the lower dose level have safely completed 28 days of treatment, with the concurrence of the investigator(s) and the PTC Therapeutics medical monitor. After all subjects have completed the 28 days of treatment at the middle dose level, the safety data for these subjects will be reviewed by the investigator(s) in coordination with the PTC Therapeutics medical monitor to determine whether to start enrollment of the highest dose level. Progression of the study to the high dose level can occur once all subjects enrolled at the middle dose level have safely completed 28 days of treatment, with the concurrence of the investigator(s) and the PTC Therapeutics medical monitor.

If a subject cannot safely continue with the full course of study medication because of dose-limiting toxicity at the lower dose level, the subject should be removed from study treatment (see Section 8). No dose reduction will be allowed for subjects enrolled at the low dose level. Appropriate follow-up (≥28 days as per protocol plan or until recovery from or stabilization of the adverse event) should be instituted.

If it is not appropriate for a subject to continue with study treatment at the middle and high dose levels because of dose-limiting toxicity, then the subject should be removed from study treatment (see Section 8), and appropriate follow-up (≥28 days as per protocol plan, or until recovery from or stabilization of the adverse event) should be instituted. However, if it is deemed that the subject can continue on study at a lower dose level, then study drug administration can be omitted, as necessary, until the adverse event resolves or stabilizes to an acceptable degree. For subjects experiencing a dose-limiting toxicity at the middle dose level, the dose of PTC124 should be reduced from the 10-, 10, and 20-mg/kg dose level to a 7-, 7- and 14-mg/kg dose level. If warranted, a further reduction to the 4-, 4-, and 8-mg/kg dose level may be considered.

For subjects experiencing a dose-limiting toxicity at the high dose level, the dose of PTC124 should be reduced from the 20-, 20, and 40-mg/kg dose level to a 15-, 15-, and 30-mg/kg dose level. The appropriate dosing chart in Appendix B provides instructions regarding the revised number of study medication sachets to be used for the 15-, 15-, and 30-mg/kg dose level. If warranted, a further reduction to the 10-, 10-, and 20-mg/kg dose level may be considered.

In general, after dose reduction, the dose should not be re-escalated. However, if further evaluation reveals that the adverse event that led to dose reduction was not study drug-related, the dose may be re-escalated to the original dose level.

Under all circumstances, the total duration of exposure to PTC124 (with or without dose modifications) should not exceed 28 days.

**Instructions for low dose and middle dose groups only**: When there is a dose-modification, the subject/caregiver should be notified of the change in dose and the appropriate clinic staff should instruct the subject/caregiver about the revised volume of reconstituted study medication corresponding to the dose to be taken/administered according to the new dose level. The appropriate dosing chart in Appendix B provides instructions regarding the volume of the reconstituted study medication to be taken by the subject at the modified dose level. Any questions regarding dose modification should be referred to the PTC Therapeutics medical monitor.

**Instructions for high dose group only**: When there is a dose-modification, the subject/caregiver should be notified of the change in dose and the appropriate clinic staff should instruct the subject/caregiver about the revised number of study medication sachets to be used per dose according to the new schedule. The appropriate dosing chart in Appendix B provides instructions regarding the revised number of study medication sachets to be used per dose according to the new schedule. Any questions regarding dose modification should be referred to the PTC Therapeutics medical monitor.

## Diet

There are no specific dietary restrictions in the study. Subjects/caregivers will be instructed to take/administer the study medication within 30 minutes after a meal; ideally the drug will be taken/administered at approximately 6-, 6-, and 12-hour intervals (eg, at ~7:00 AM after breakfast, ~1:00 pm after lunch, and at ~7:00 PM after dinner).

## Concomitant and Supportive Therapy

While conventional supportive therapies will be permitted, efforts will be made to avoid use of concomitant medications that might confound interpretation of study results. In particular, investigators will be urged, when it is clinically appropriate, to substitute other antibiotics for systemic aminoglycosides in subjects who require treatment for serious infections.

In authorizing the use of any drug other than study medication, the investigator should consider the clinical situation, the potential for masking symptoms of a more significant underlying event, and whether use of the drug will compromise the outcome or integrity of the study. In this study, collection of concomitant medication information will focus on the use of supportive care drugs. Drugs of particular interest include corticosteroids and antibiotics (especially aminoglycosides).

Subjects should avoid using alcohol or tobacco products, herbal remedies, or any self-prescribed or over-the-counter drugs at any time during the study period. Upon entering the study, subjects will be instructed about the importance of avoiding these products and of the need to inform the clinic staff if such products are used.

Any concomitant drugs (prescribed or over-the-counter) utilized during the course of the study and the reason for their use will be recorded in the source documents and on the electronic data entry forms (see Section 10.4).

## Study Drug Administration Rationale

The study is designed to maintain subject safety while enhancing the efficiency of dose-ranging in this limited subject population. By first evaluating a lower dose in a limited number of subjects, safety and PK can be profiled in the pediatric age group. Once safety and adequate exposure are documented at the lower dose, enrollment of the majority of the subjects at the middle and high dose maximizes the potential to observe a positive efficacy signal. The planned 4-week duration of therapy builds on the preclinical efficacy data [Barton 2005], and the safety data from the 28-day rat and dog toxicology studies. This 4-week duration of treatment is an appropriate extension from the preceding 2-week Phase 1 multiple-dose trial.

The proposed doses are based on assessments of the available Phase 1 and Phase 2 safety and PK data considered in conjunction with long-term nonclinical toxicology and exposure information. However, the evolving Phase 2 exposure data will be considered in conjunction with the safety data. If these data in subjects with DMD indicate that doses higher than those proposed in this study should be evaluated, the highest dose administered will have a projected exposure no greater than that associated with the highest dose used in the multiple-dose study (50 mg/kg BID). If it is deemed appropriate to evaluate different doses than those proposed, a protocol amendment will be submitted to the IRB for approval.

Within the known variability of PTC124 PK, the dose assignment plan is designed to achieve reasonable accuracy of dosing and simplicity of drug administration. The method favors adequate drug exposure while conserving a substantial safety margin.

Clinic staff will administer the study medication during the time the subject is confined to the clinic so that the PK measurements are taken at the appropriate time intervals relative to dosing. Instruction, provision of written information, and close follow-up by clinic staff are intended to maximize PTC124 reconstitution accuracy and dosing compliance.

Preclinical studies indicate that sustained exposure to PTC124 may be required for efficacy; the target pharmacological effect is rapidly reversed following drug washout. Because the t_1/2_ of PTC124 is relatively short, sustaining plasma concentrations will require divided dosing. Interim Phase 2 results indicate that TID PTC124 administration – using 2 smaller doses given at 6-hour intervals during the day and a larger dose given at a 12-hour interval overnight (eg, at 7:00 AM, 1:00 PM, and 7:00 PM) with food is safe and is associated with excellent compliance. This administration plan also capitalizes on the apparent food effect in maintaining PTC124 plasma concentration at 12 hours without increasing C_max_, AUC, or toxicity.

The dose modification provisions are designed to balance a primary concern for subject safety with the potential for observing pharmacodynamic activity in circumstances under which a subject experiencing an adverse event may still be able to continue on therapy at an intermediate PTC124 dose level.

# Schedule of Events and study parameters

## Schedule of Events

The proposed types and timing of data to be collected and recorded are described in Table 2 below. Please see Section 6.2 below for cross-referenced explanations of the study procedures described in the table.

| Table 2. DMD Dose-Ranging Phase 2a Study Schedule of Events | | | | | | | | | | |
| --- | --- | --- | --- | --- | --- | --- | --- | --- | --- | --- |
| **Protocol Activities** | **Pretreatment Period {7.2.1}** | **Treatment Period**  **{7.2.1}** | | | | | | **End of Study Follow-up Period**  **{7.2.1}** | | **Long- Term Follow-up** |
|  |  | **Day** | | | | | | **Day** | | **Up to 5 Years** |
|  | **Screening  Day -21 to -1** | **1** | **7**  **(± 1)** | **14**  **(± 1)** | **21**  **(± 1)** | **27**  **(± 1)** | **28** | **42 (± 1)** | **56**  **(± 1)** |  |
|  | **Visit** | | | | | | | | |  |
|  | **1** | **2** | **3** | **4** | **5** | **6** | | **7** | **8** |  |
| Informed Consent | X |  |  |  |  |  |  |  |  |  |
| Medical History | X |  |  |  |  |  |  |  |  |  |
| Dystrophin Gene Sequencing {7.2.2} | X |  |  |  |  |  |  |  |  |  |
| Hepatitis and HIV Screen {7.2.3} | X |  |  |  |  |  |  |  |  |  |
| Vital Signs {7.2.4} | X | X |  |  |  |  | X |  |  |  |
| Height and Weight {7.2.5} | X |  |  |  |  |  | X |  | X |  |
| Physical Examination {7.2.5} | X |  |  |  |  |  | X |  | X |  |
| Hematology Lab Assessment {7.2.6} | X | X | X | X | X | X |  | X | X |  |
| Biochemistry Lab Assessment {7.2.7} | X | X | X | X | X | X |  | X | X |  |
| Coagulation Lab Assessment {7.2.8} | X |  |  |  |  | X |  |  |  |  |
| Urinalysis {7.2.9} | X |  |  |  |  | X |  |  | X |  |
| 12-Lead ECG {7.2.10} | X |  |  |  |  | X |  |  | X |  |
| PTC124 Administration {7.2.11} |  | X--------------------------------------------X | | | | | |  |  |  |
| PTC124 Compliance {7.2.12} |  |  | X | X | X | X |  |  |  |  |
| Adverse Events {7.2.13, 7.2.14} | X | X | X | X | X | X | X | X | X |  |
| Concomitant Medications {7.2.15} | X | X | X | X | X | X | X | X | X |  |
| Muscle and Skin Biopsy {7.2.16} | X |  |  |  |  |  | X |  |  |  |
| Upper/Lower Extremity Myometry {7.2.17} | X |  |  |  |  | X |  |  | X |  |
| Timed Function Tests {7.2.18} | X |  |  |  |  | X |  |  | X |  |
| Pharmacokinetics for PTC124 {7.2.19} |  | X |  |  |  | X-------X | |  |  |  |
| Long-term Follow-up {7.2.20} |  |  |  |  |  |  |  |  |  | X |
| **Abbreviations:** ECG = electrocardiogram, HIV = human immunodeficiency virus | | | | | | | | | | |
|  | | | | | | | | | | |

## Explanation of Study Procedures

### Pretreatment, Treatment, and Follow-up Periods

Screening evaluations for the study will be performed at the clinical research facility as needed. Study participants will report to the clinic on Day -1 (the day prior to first dose administration). Subjects will remain in the clinic until released by the investigator on the morning of Day 2, after the last (12-hour) PK sample following the Day 1 evening dose is collected, all the study-related procedures have been completed, and the subject/caregiver has been instructed regarding drug storage, reconstitution, and administration. Subjects will be contacted either via telephone or through a visit to the clinical research facility on Days 7, 14, and 21 for collection of information regarding adverse events, concomitant medications, and compliance. Participants will also report to the clinic on Day 26, for performance of functional tests and PK sampling on Day 27 and muscle biopsy on Day 28. Subjects will remain in the clinic until released by the investigator on Day 28 or Day 29.

Subjects will be either contacted via telephone or through a visit to the clinical research facility on Day 42 for collection of information regarding adverse events and concomitant medications, and may have the necessary Day 42 hematology and biochemistry studies done at a local laboratory. Subjects must return for follow-up assessment at the clinical research facility on Day 56.

As noted in Table 2, study-related procedures and information collection should be accomplished within ±1 day of the stipulated time.

### Dystrophin Gene Sequencing

Blood for dystrophin gene sequencing will be collected during Screening for documentation of the presence of a nonsense mutation. This sample will be shipped to the University of Utah, Salt Lake City, UT (see Study Manual for details). A subject who has already had gene sequencing performed by the University of Utah does not need a repeat test performed. A subject who has documentation of a nonsense mutation by other methods need not wait for confirmatory results to start study therapy as long as a blood sample has been sent to the University of Utah.

The blood sample collected for dystrophin gene sequencing will be destroyed after the test is completed and a final report is generated by the University of Utah genetics laboratory. No other DNA/gene sequencing will be performed.

### Hepatitis and HIV Screen

Screening tests include hepatitis B surface antigen, hepatitis C antibody, and HIV antibody.

### Vital Signs

Vital signs (pulse, respiratory rate, blood pressure, and temperature) while resting will be monitored once during Screening, just prior to first dose of PTC124 on Day 1, at approximately 1 hour after the first dose on Day 1, and 1 hour after the last dose (ie, evening dose) of PTC124 on Day 28 (last day of treatment with PTC124). If the subject is discharged from the clinic on Day 28 prior to administration of the last dose of PTC124, the subject/caregiver should be given the remaining doses and instructed to complete the dosing at home. In this situation, the vital signs should be collected in relation to the last dose of PTC124 administered in the clinic.

### Height, Weight, and Physical Examination

Height (in cm) will be measured once, at Screening. Weight (in kg) will be measured during Screening, and on Day -1, Day 28 (last day of treatment with PTC124), and Day 56 of the study.

Physical examination will be conducted during Screening, on Day 28 of the treatment period, and at the end of study visit on Day 56 and during the study as clinically indicated.

### Hematology Laboratory Assessment

Hematology laboratory assessments will include white blood cell count with differential, hemoglobin, hematocrit, other red cell parameters, and platelet count. These parameters will be monitored during Screening; on Day 1 (prior to first dose of PTC124 unless the Screening assessment was done within 7 days prior to dosing), Day 7, Day 14, Day 21, and Day 27 during the treatment period; and on Day 42 and Day 56 during the follow-up period.

If the patient is not seen in the clinic on Day 7, Day 14, Day 21, and/or Day 42, the blood sample for the assessment of hematological parameters can be drawn at a local laboratory closer to the patient’s home.

### Biochemistry Laboratory Assessment

Biochemistry laboratory assessments will include sodium, potassium, chloride, bicarbonate, blood urea nitrogen, creatinine, calcium, phosphorus, uric acid, glucose, total protein, albumin, globulin, albumin:globulin ratio, bilirubin (direct and indirect), AST, ALT, GGT, CK, LDH, alkaline phosphatase, ammonia, total cholesterol, triglycerides, low-density lipoprotein, and high-density lipoprotein. These parameters will be monitored during Screening; on Day 1 (prior to first dose of PTC124 unless the Screening assessment was done within 7 days prior to dosing), Day 7, Day 14, Day 21, and Day 27 during the treatment period; and on Day 42 and Day 56 during the follow-up period.

If the patient is not seen in the clinic on Day 7, Day 14, Day 21, and/or Day 42, the blood sample for the assessment of biochemistry parameters can be drawn at a local laboratory closer to the patient’s home.

### Coagulation Laboratory Assessment

Coagulation laboratory assessments will include prothrombin time (PT), activated partial thromboplastin time (aPTT), and International Normalized Ratio (INR). These parameters will be monitored during Screening (prior to muscle biopsy procedure) and on Day 27 (prior to muscle biopsy procedure on Day 28) during the treatment period.

### Urinalysis

Urinalyses will include dipstick analysis for pH, specific gravity, glucose, ketones, blood, protein, urobilinogen, bilirubin, and microscopic examination. These parameters will be monitored during Screening, on Day 27 during the treatment period, and on Day 56 during the follow-up period.

### 12-Lead ECG

A 12-lead ECG will be obtained during Screening, on Day 27 during the treatment period, and on Day 56 during the follow-up period.

### PTC124 Administration

PTC124 administration will be started on Day 1 in the clinic and continued on a TID (6-, 6-, and 12-hour intervals) schedule daily for 28 days. Each subject will receive 84 doses of PTC124 during the study. PTC124 will be supplied to the subject/parent/caregiver at the time of first discharge from the clinic on Day 2 of the study. Clinic staff will administer the study medication during the time the subject is confined to the clinic.

### PTC124 Compliance

**Low-dose and Mid-dose level Compliance Measurement:** PTC124 compliance will be assessed using a Patient Study Drug Compliance Log and a Medication Administration Record, which will document the return of any unused drug for compliance assessments. On Day 1, while the subject is in the clinic, the study staff will demonstrate how to record the necessary drug use information in the log. In addition, at the Day 7, Day 14, and Day 21 visits (or telephone contact if the subject does not visit the clinic on the scheduled day), the clinic staff will also discuss and resolve any issues that the subject/parent/caregiver may have with the drug preparation procedures. Subjects/caregivers will be asked to record the date and time of day (ie, breakfast, lunch, or dinner) at which the drug was taken, and will be questioned for the reason for any missed doses (see Study Manual). Subjects/caregivers should return all bottles (including used and partially used bottles, and unused opened or sealed bottles) to the study site on Day 27 for full compliance verification. Subject/caregiver will also be asked to record the date and time (breakfast, lunch, or dinner) of use of the study medication bottle on the PTC124 bottle itself. Clinic staff will record the study medication dosing information, including the exact clock time of each dose, during the time the subject is confined to the clinic. Any discrepancies between the Patient Study Drug Compliance Log and the Medication Administration Record will be noted.

**High Dose-level Compliance Measurement:** PTC124 compliance will be assessed using a Patient Study Drug Compliance Log and a Medication Administration Record, which will document the return of used sachets and any unused drug sachets for compliance assessments. On Day 1, while the patient is in the clinic, the study staff will demonstrate how to record the necessary drug use information in the log. This log will then be completed by the patient/guardian each day for each dose and will be returned to the study personnel at each visit. . In addition, at the Day 7, Day 14, and Day 21 visits (or telephone contact if the subject does not visit the clinic on the scheduled day), the clinic staff will also discuss and resolve any issues that the subject/parent/caregiver may have with the drug preparation procedures. Subjects/caregivers will be asked to record the date and time of day (ie, breakfast, lunch, or dinner) at which the drug was taken, and will be questioned for the reason for any missed doses (see Study Manual). Subjects/caregivers should return all sachets (including used and partially used sachets, and unused opened or sealed sachets) to the study site on Day 27 for full compliance verification. Clinic staff will record the study medication dosing information including the actual clock time of each dose during the time the patient is confined in the clinic. Any discrepancies between the Patient Study Drug Compliance Log and the Medication Administration Record will be noted.

### Adverse Events

Any adverse events must be assessed and documented at each scheduled clinic visit, ie, during Screening, on Day 1 (prior to first dose of PTC124), on Day 7, Day 14, Day 21, at other times during the study treatment period if adverse events are reported by the subject, on Day 28 (last day of treatment with PTC124), and on Day 42 and Day 56 during the follow-up period. If the subject does not visit the clinic on the scheduled day, a telephone call to the subject/caregiver is required to assess any adverse events. Subjects must be followed for adverse events for 28 days after the last dose of study treatment, or until any drug-related adverse events and/or ongoing serious adverse events have resolved or become stable, whichever is later.

### Follow-up of Adverse Event or Abnormal Laboratory/ECG Findings

Laboratory or ECG results with significantly abnormal values should be repeated for verification. Additional tests and other evaluations required to establish the significance or etiology of an abnormal result, or to monitor the course of an adverse event, should be obtained when clinically indicated. If a clinically significant adverse event or abnormal result is observed that is not resolved by the final study visit, repeat evaluations should be performed to document resolution or stabilization of the abnormality.

### Concomitant Medications

Concomitant medication information will be collected and documented at each scheduled clinic visit, ie, during Screening; on Day 1 (prior to first dose of PTC124), Day 7, Day 14, Day 21 during the study treatment period if concomitant medication use is reported by the subject, on Day 28 (last day of treatment with PTC124), and on Day 42 and Day 56 during the follow-up period. If the subject does not visit the clinic on the scheduled day, a telephone call to the subject/parent/caregiver is required to assess concomitant medication use. All concomitant drug use should be reported; drugs of particular interest include corticosteroids and antibiotics (especially aminoglycosides).

### Muscle and Skin Biopsies

Biopsy of the EDB/TA muscle and overlying skin from one foot will be performed under local anesthesia and conscious sedation (in some cases, general anesthesia may be required) during the Screening period, and from the other foot on Day 28 (last day of treatment with PTC124) of the study. The biopsy procedure will be performed using standardized techniques [Stedman 2000] (see Study Manual). The entire muscle belly (whenever possible) will be removed in the procedure.

At the time of collection of the biopsy during the Screening period, the muscle specimen will be divided into at least 3 fragments and the biopsy specimen collected on Day 28 should be divided into at least 2 fragments. The fragments should be prepared for analyses as per the procedures described in the Study Manual. All sample containers will be clearly labeled in a fashion that identifies the subject and the collection date. Labels will be fixed to the sample containers in a manner that will prevent the label from becoming detached.

Samples should be shipped for analysis/culture/central review immediately after the procedure is performed (see Study Manual for collection, processing, and shipping details). To protect against sample loss, samples should only be shipped overnight on Monday, Tuesday, or Wednesday using an overnight priority courier, and PTC Therapeutics should be notified at the time of shipping that samples are in transit. Upon receipt of the shipment, the review site will provide an e-mail verification to the treatment site and the sponsor that the samples were received.

Under the direction of Carsten G. Bonnemann, MD at The Children’s Hospital of Philadelphia, muscle sections will be processed and immunostained to detect muscle membrane-localized dystrophin, various sarcoglycans, and dystroglycan; dystrophin mRNA will also be quantified. For detection of dystrophin, 3 commercially available antibodies that recognize the C‑terminus, the N-terminus, and the rod domain of the protein will be employed. For detection of the sarcoglycan and dystroglycan complex, commercially available antibodies against α-, β-, γ-, and δ-sarcoglycan, and β-dystroglycan will be used whenever possible. Epifluorescence microscopy will be used in the analysis; images will be captured by CCD camera, after normalization of the fluorescence intensity against a normal muscle specimen. Images will be stored digitally and preserved for future review, and final evaluation at the completion of the study. Tissues will also be processed for detection of dystrophin, the sarcoglycans, and β-dystroglycan by Western blotting using the same antibodies. Microscopic images will be captured and preserved for future review, and for final evaluation at the completion of the study. Remaining muscle tissue samples will be preserved for confirmatory assays of mRNA and proteins involved in DMD.

From the muscle and skin biopsy obtained during the Screening period, tissues will also be processed in order to establish primary muscle cell cultures at Dr. Bonneman’s laboratory, and to establish dermal fibroblast cultures under the direction of Lee Sweeney, PhD, at The Children’s Hospital of Philadelphia. Culture conditions will be established to differentiate the dermal fibroblasts into muscle cells by transfection of the cells with a Myo-D-producing expression construct [Wang 2001]. These muscle and fibroblast cultures will be used to detect dystrophin and dystrophin-associated protein production in response to in vitro PTC124 exposure. Immunostaining and Western blotting will be employed for protein detection. Microscopic images will be captured and preserved for future review, and for final evaluation at the completion of the study. Remaining skin and muscle tissue samples will be preserved for confirmatory assays of mRNA and proteins involved in DMD.

### Upper and Lower Extremity Myometry

To the extent possible (depending on the subject’s baseline functional status), upper and lower extremity myometry will be performed using a hand-held myometer following standardized procedures [Beenakker 2001, Hyde 2001] (see Study Manual). Muscle groups to be evaluated will include hip abductors, knee extensors, elbow flexors and extensors, and hand grip. Bilateral assessments should be done, and three measurements should be recorded from each muscle group on each side if possible. These parameters will be monitored during Screening, on Day 27 during the treatment period, and on Day 56 during the follow-up period. During the Screening and treatment periods, the myometry procedures should be performed prior to the muscle biopsy. All attempts should be made to complete the myometry assessments prior to starting the PK sample collection on Day 27 as the needle inserted in the arm for repeated blood-draw can lead to altered performance effort by the patient.

### Timed Function Tests

In ambulatory patients, timed function tests will include time taken to stand from a supine position, time taken to walk 10 meters, and time taken to climb 4 standard-sized stairs [Mendell 1989, Griggs 1991] (see Study Manual). For non-ambulatory patients, timed function tests should not be carried out. These parameters will be monitored during Screening, on Day 27 during the treatment period, and on Day 56 during the follow-up period. During the Screening and treatment periods, the timed function tests should be performed prior to the muscle biopsy. All attempts should be made to complete the timed function tests prior to starting the PK sample collection on Day 27 as the needle inserted in the arm for repeated blood-draw can lead to altered performance effort by the patient.

### Blood for Analysis of PTC124 Pharmacokinetics and Metabolism

Blood for PK assessments will be collected on Days 1 through 2 and Days 27 through 28 during the treatment period.

Each subject will be admitted to the clinical research center no later than the evening of Day -1 in preparation for PK sample collection on Days 1 and 2. The subject will again be admitted to the clinical research center no later than the evening of Day 26 in preparation for PK sample collection on Days 27 and 28.

Relative to the Day 1 and Day 27 dosing, blood samples for PTC124 PK assessments will be collected immediately pre-dose and at approximately 1, 2, 3, and 4 hours after administration of the breakfast (~7:00 AM) dose; immediately pre-dose and at approximately 1, 2, 3, and 4 hours after administration of the lunch (~1:00 PM) dose; and immediately pre-dose and at approximately 1, 2, 3, 4, and 12 hours after administration of the dinner (~7:00 PM) dose (continuing into Day 2 or Day 28, respectively, see Table 3). The actual sample collection times will be recorded. A subject may have a heparinized venous catheter placed for sample collection in order to avoid repeated needle sticks. If a catheter is used, at least 2 mL of blood should be removed and discarded prior to each sample collection in order to avoid heparin contamination of the sample. All attempts should be made to collect the blood samples at, or within ±5 minutes of, the scheduled time. The timing of the blood draw will be in relation to the PTC124 dosing time and not the time of the preceding meal.

| Table 3. Schedule of Pharmacokinetic Sample Collection | | | | | | | |
| --- | --- | --- | --- | --- | --- | --- | --- |
| **Day** | | **Dose Time** | **Time** | | | | |
|  |  |  | **Predose** | **Postdose (hrs)** | | | |
|  |  |  | **0** | **1** | **2** | **3** | **4** |
| 1 | | 7:00 AM | X | X | X | X | X |
|  |  | 1:00 PM | X | X | X | X | X |
|  |  | 7:00 PM | X | X | X | X | X |
| 2 | | 7:00 AM | X* |  |  |  |  |
|  |  | 1:00 PM |  |  |  |  |  |
|  |  | 7:00 PM |  |  |  |  |  |
| 27 | | 7:00 AM | X | X | X | X | X |
|  |  | 1:00 PM | X | X | X | X | X |
|  |  | 7:00 PM | X | X | X | X | X |
| 28 | | 7:00 AM | X* |  |  |  |  |
|  |  | 1:00 PM |  |  |  |  |  |
|  |  | 7:00 PM |  |  |  |  |  |
| * | Approximately 12 hours after the last dose on Day 1 or Day 27 | | | | | | |
|  | | | | | | | |

Each sample will comprise 2 mL of venous blood drawn into a 5-mL Vacutainer® or equivalent tube with K_3_-EDTA as the anticoagulant. Immediately after collection, the tube should be gently inverted 8 to 10 times to mix the anticoagulant with the blood sample. The tube should be stored upright on ice until centrifugation; centrifugation and sample processing should be performed within 1 hour of sample collection. The plasma fraction should be separated by placing the collection tube into a refrigerated centrifuge (4 to 8^o^C) in a horizontal rotor (with a swing-out head) for a minimum of 15 minutes at 1500 to 1800 relative centrifugal force (RCF). The plasma fraction will be withdrawn by pipette and divided into 2 polypropylene freezing tubes (with each tube receiving approximately equal aliquots). All sample collection and freezing tubes will be clearly labeled in a fashion that identifies the subject, the study period, and the collection date and time. Labels will be fixed to freezing tubes in a manner that will prevent the label from becoming detached after freezing. After processing, samples should be placed into a freezer at approximately –20^o^C (or lower).

To protect against sample loss, the samples will be divided into 2 shipments, each containing 1 aliquot of plasma for each time point. The first aliquots of the samples should be shipped within 24 hours after collection of the last sample on Day 2 (12-hour sample) and again after collection of the last sample on Day 28 (12-hour sample). The aliquots of the samples from all timepoints within a collection period should be shipped together. Prior to shipping, the samples will be packed in thermal insulated containers with sufficient dry ice to ensure they remain frozen and are protected from breakage during shipment. Samples will be shipped overnight via priority courier to SFBC Analytical Laboratories. After receipt of verification that the first shipment was received by the analytical facility, the second shipment will be shipped (see Study Manual for shipping details).

Analyses of the PK samples for PTC124 will be performed at SFBC Analytical Laboratories using a validated HPLC-MS-MS method. Since this is the first study of PTC124 in the pediatric population and since profiling of PTC124 metabolism is currently ongoing, remaining portions of plasma samples collected for PK analysis will be preserved for PTC124 metabolite analysis.

### Long-term Follow-up

Data on patient survival and on the occurrence of new non-DMD-related health problems (eg, chronic hepatic abnormalities, endocrine disorders, tumors, etc) will be collected from all patients enrolled in the study who receive at least one dose of PTC124. The information will be gathered during a routine clinic visit or other contact with the patient, or via telephone. As measured from the start of study treatment, these data will be collected at approximately 6-month intervals during Year 1 and Year 2, and at yearly intervals during Years 3, 4, and 5. These data will be collected in the source documents (eg, patient medical record) and transcribed onto a paper case report form that will be transmitted to the data management CRO (Innovative Analytics, Kalamazoo, MI) for entry into the database. Any unsuccessful efforts to contact the patient (eg, dates of unanswered phone calls, return of certified letter sent to patient’s home, etc.) will be documented in the patient’s source documents.

## Blood Collection Summary

Information regarding the types and amounts of blood samples to be collected in the study is provided in Table 4.

| Table 4. Blood Drawing Requirements | | | | | | | |
| --- | --- | --- | --- | --- | --- | --- | --- |
| **Test** | **Sample Type** | **Tube Type** | **Tube Top (Color)** | **Tube Size (mL)** | **Blood Per Tube (mL)** | **Number**  **of Tubes** | **Total Blood (mL)** |
| Dystrophin Gene Sequencing | Blood | ACDA | Yellow | 10 | 5 | 1 | 5 |
| Hematology Assessment | Plasma | EDTA | Purple | 5 | 2 | 8 | 16 |
| Biochemistry Assessment (Hepatitis and HIV Screen) | Serum | Clot | Red | 10 | 8 | 8 | 64 |
| Coagulation Assessment | Blood | EDTA | Purple | 10 | 5 | 2 | 10 |
| Blood for PTC124 PK | Plasma | K_3_-EDTA | Lavender | 5 | 2 | 32 | 64 |
| **Total** |  |  |  |  |  |  | 159 |
| **Abbreviations:** ACDA = acid citrate dextrose solution ‘A’, EDTA = ethylenediaminetetraacetate,  HIV = human immunodeficiency virus, PK = pharmacokinetics | | | | | | | |
|  | | | | | | | |

## Study Parameter Selection Rationale

The planned study assessments and timing have been selected as appropriate for screening of subjects, for evaluation of drug activity, for dose modification during the study, for determination of treatment-related or disease-related toxicities, and for characterization of drug exposure. The scheduling of tests is designed to balance collection of a complete efficacy and data set with subject tolerance of study procedures. For discussion of the rationale for endpoint selection, see Section 1.5.

Because PTC124 is a new chemical entity with a novel mechanism of action, the FDA has requested that patients receiving the drug undergo long-term follow-up in order to build a safety database regarding the potential for any late toxicities.

# Adverse Event Assessments

## Adverse Event Definitions

### Adverse Events

An adverse event is any untoward medical occurrence in a trial subject who is administered a drug or biologic (medicinal product) or who is using a medical device; the event does not necessarily have a causal relationship with study drug administration or device usage. For this protocol, untoward medical occurrences that should be reported as adverse events include the following:

- All adverse events that are suspected to be due to study medication
- All adverse events that are suspected to be due to concomitant medication
- Apparently unrelated illnesses, including the worsening of a preexisting illness
- Injury or accidents. Note that if a medical condition is known to have caused the injury or accident (a fall secondary to dizziness), the medical condition (dizziness) and the accident (fall) should be reported as 2 separate adverse events. The outcome of the accident (hip fracture secondary to the fall) should be recorded in source documents.
- Abnormalities in physiological testing or physical examination findings that require clinical intervention or further investigation (beyond ordering a repeat [confirmatory] test)
- Laboratory or ECG abnormalities that require clinical intervention or further investigation (beyond ordering a repeat [confirmatory] test) unless they are associated with an already reported clinical event. Laboratory abnormalities associated with a clinical event (eg, elevated liver enzymes in a subject with jaundice) should be captured in the source documents. Laboratory abnormalities not requiring clinical intervention or further investigation will be captured as part of overall laboratory monitoring, and should not be reported as adverse events.
- A preexisting condition (eg, allergic rhinitis) must be noted on the appropriate baseline electronic data entry form (see Section 10.4) but should not be reported as an adverse event unless the condition worsens or episodes increase in frequency during the adverse event reporting period. Diagnostic and therapeutic non-invasive and invasive procedures, such as surgery, should not be reported as adverse events. However, the medical condition for which the procedure was performed should be reported if it meets the definition of an adverse event. For example, an acute appendicitis that begins during the adverse event reporting period should be reported as the adverse event and the resulting appendectomy should be recorded in the source documents. If a surgical procedure was planned prior to entry into the trial, and the surgery is not performed because of a worsening of a baseline condition, this should not be reported as an adverse event. Note that, as described in Section 7.1.2, any inpatient hospitalization occurring as the consequence of an adverse event during the study period should be reported as a serious adverse event.

### Serious Adverse Events

In this protocol, an adverse event that results in 1 or more of the following outcomes is classified as serious:

- Death
- Life-threatening situation (ie, with an immediate risk of death)
- Inpatient hospitalization or prolongation of existing hospitalization. Note that a hospitalization for protocol-specified activities or for pre-planned procedures should not be reported as a serious adverse event.
- Persistent or significant disability/incapacity
- Malignancy
- Other important untoward medical occurrences that may not result in death, may not be life-threatening, and do not require hospitalization, but which may be considered serious when, based upon appropriate medical judgment, they may jeopardize the subject or may require medical or surgical intervention to prevent any of the outcomes listed above. Examples of such medical events include allergic bronchospasm requiring intensive treatment administration in an emergency room or at home, blood dyscrasias or convulsions that do not result in inpatient hospitalization, or the development of drug dependency or drug abuse.

### Unexpected Adverse Events

Unexpected adverse events are defined as those events that are not described in the protocol, Investigator Brochure, or consent form as being previously reported with administration of the study drug, or that are symptomatically and pathophysiologically related to a known toxicity but differ because of greater severity or specificity.

Table 5 provides a summary of the safety profile of PTC124 based on a preliminary review of the safety data from the Phase 1 single-dose and multiple-dose studies in healthy volunteers and ongoing Phase 2 studies in patients with CF and DMD.

| Table 5. Preliminary PTC124 Safety Profile | | |
| --- | --- | --- |
| **Relationship to PTC124** Ф | | **Adverse Event or Abnormal Laboratory Value*** |
| Probable | | Nausea^†^, vomiting^†^, diarrhea^†^, dysuria, mucosal burning sensation (throat), dry throat, headache, increased ALT, increased AST, increased LDH^‡^, increased aldolase^‡^ |
| Possible | | Dizziness, drowsiness†, tremors†, headache§, asthenia, myalgia†, arthralgia†, (musculoskeletal) chest pain, increased thirst, dry mouth, scratchy throat, increased cough, abdominal pain/discomfort§, constipation/intestinal obstruction§, nausea§, vomiting, diarrhea, flatulence, nasal congestion, rhinorrhea, ocular hyperemia, petechiae, pruritus, bladder pain/dysuria†, increased serum CK^‡^ |
| Unlikely | | Eye pain, eye swelling, eye irritation, allergies, nipple pain^†^, breast tenderness, rash, lymph node pain, swollen glands§, fall, pain from study procedure, skin infection^†^, tremor, chills, headache†, myalgia†, arthralgia^†^, asthenia†, chest pain, abdominal pain†, sore throat§, aphonia, sinusitis†, increased cough§, nasal congestion, dyspnea§, pneumonia§, upper respiratory infection, hemoptysis, CF exacerbation^§^, pyrexia†, cold, sunburn†, vomiting, flatulence, diarrhea, , increased triglycerides, hyperglycemia† |
| * | Unless noted, the greatest severity of clinical signs and symptoms reported has been Grade 1 (mild) based on the CTCAE, Version 3.0. Frequency of an adverse event or laboratory abnormality has not been included in this table. | |
| † | These clinical symptoms were described as Grade 2 (moderate). | |
| ‡ | Elevations in CK, LDH, and aldolase as high as Grade 4 were reported in the Phase 1 clinical experience with PTC124. When first reported by the investigator, these were considered to be possibly or probably drug related. However, more experience has indicated that these events are likely attributable to muscle stress injury arising from heavy physical exertion rather than to PTC124. | |
| § | This clinical symptom was described as Grade 3 (severe). | |
| Ф | Within each relationship category, the highest grade of severity is reported | |
| **Abbreviations:** ALT = alanine aminotransferase, AST = aspartate aminotransferase, CF = cystic fibrosis, CK = creatine kinase, LDH = lactate dehydrogenase; CTCAE = Common Terminology Criteria for Adverse Events | | |
|  | | |

## Eliciting Adverse Event Information

The investigator is to report all directly observed adverse events and all adverse events spontaneously reported by the trial subject or parent/guardian in case of a child. In addition, each trial subject will be questioned about adverse events at each scheduled clinic visit after study drug administration or during each telephone contact with the subject or parent/guardian in case of a child. The type of question asked should be open-ended, eg, *“Has your child had any new health problems?”* or a similar type of query.

## Adverse Event Recording

All adverse events (both serious and nonserious) that occur in subjects during the adverse event reporting period defined in Section 7.5 must be recorded, whether or not the event is considered drug-related. In addition, any known untoward event that occurs subsequent to the adverse event reporting period that the investigator assesses as possibly related to the investigational drug/product should also be recorded as an adverse event.

All adverse events are to be recorded in the source documents and on the electronic data entry forms (see Section 10.4) using concise medical terminology, and, whenever possible, terms contained in MedDRA. In addition, the following information should be recorded:

- Time of last dose prior to onset of event
- Onset date and time
- Resolution date and time, or date and time of death
- Severity of the event (see Section 7.6)
- Action taken
- Outcome of the event
- Relationship to study drug (see Section 7.7)
- Indication of whether the event is serious or nonserious; if serious, the reason for this classification

## Adverse Event Reporting Requirements

Classification of the event as serious or nonserious determines the reporting procedures to be followed. Reporting requirements for adverse events are summarized in Table 6 below.

| Table 6. Reporting Requirements For Adverse Events | | |
| --- | --- | --- |
| **Classification** | **Reporting Time** | **Reporting Action** |
| **Serious** | Within 24 hours | Telephone call to PTC Medical Monitor |
|  | Within 24 hours | Fax report on designated Serious Adverse Event Form to PTC Therapeutics and to IRB |
|  | Within 5 days | Submit photocopies of relevant source documents (eg, progress notes, nurses’ notes, laboratory and diagnostic test results, discharge summaries) to PTC Therapeutics, and ensure that data entry has been completed in the electronic data entry forms for adverse events, medical history, and concomitant drug/therapy |
|  | Per electronic data entry procedure | Record and submit information on appropriate electronic data entry forms |
| **Nonserious** | Per electronic data entry procedure | Record and submit information on appropriate electronic data entry forms |
| **Abbreviations:**  IRB = Institutional Review Board | | |
|  | | |

For serious adverse events, in addition to completing the Adverse Event section(s) of the electronic data entry forms (see Section 10.4), the provided (paper) Serious Adverse Event Form must also be completed. Where the same data are collected, the forms must be completed in a consistent manner. For example, the same adverse event term should be used on both forms.

For serious adverse events, the contact information for initial reporting of events is the PTC Therapeutics medical monitor.

All other documentation pertaining to the serious adverse event must also be forwarded to the PTC Therapeutics medical monitor within 5 working days.

As appropriate, PTC Therapeutics will submit an Investigational New Drug (IND) safety report and will notify the investigator of any serious and unexpected adverse events that have required expedited reporting to the FDA and/or other health authorities.

In the rare event that the investigator does not become aware of the occurrence of a serious adverse event immediately (for example, if a subject initially seeks treatment elsewhere), the investigator is to report the event within 24 hours after learning of it and to document his/her first awareness of the adverse event.

## Adverse Event Reporting Period

The first day of adverse event reporting will coincide with the day the informed consent is signed. For this study, the adverse event reporting period ends 28 days after the last administration of the study drug or until all drug-related adverse events have resolved or become stable, whichever is later.

## Grading of Adverse Event Severity

The severity of adverse events will be graded using the CTCAE, Version 3.0 (see the Study Manual). For each event, the highest severity grade attained should be reported.

If a CTCAE criterion does not exist, the investigator should use the grades or adjectives defined in Table 7.

| Table 7. Grading of Adverse Event Severity (Events not Graded in CTCAE) | | |
| --- | --- | --- |
| Grade | Adjective | Description |
| Grade 1 | Mild | Sign or symptom is present, but it is easily tolerated, is not expected to have a clinically significant effect on the subject’s overall health and well being, does not interfere with the subject’s usual function, and is not likely to require medical attention |
| Grade 2 | Moderate | Sign or symptom causes interference with usual activity or affects clinical status, and may require medical intervention |
| Grade 3 | Severe | Sign or symptom is incapacitating or significantly affects clinical status and likely requires medical intervention and/or close follow-up |
| Grade 4 | Life-threatening | Sign or symptom results in a potential threat to life |
|  | | |

Note the distinction between the seriousness and the severity of an adverse event. Severity is a measure of intensity; thus, a severe reaction is not necessarily a serious reaction. For example, a headache may be severe in intensity, but would not be classified as serious unless it met one of the criteria for serious events listed in Section 8.1.2 above.

## Describing Relationship to Study Drug

An evaluation of whether an adverse event is potentially related or is unrelated to the study drug should be provided by the investigator based on the considerations outlined in Table 8 below.

| Table 8. Relationship of Study Drug To Adverse Event | |
| --- | --- |
| Relationship | Description |
| **Probable** | A clinical event in which a relationship to the study drug seems probable because of such factors as consistency with known effects of the drug; a clear temporal association with the use of the drug; improvement upon withdrawal of the drug; lack of alternative explanations for the event; or other factors. |
| **Possible** | A clinical event occurring coincident with administration of the study drug and which is not likely to be explained by concurrent disease or other drugs or chemicals. Information on drug withdrawal may be lacking. |
| **Unlikely** | A clinical event with a temporal relationship to study drug administration that makes a causal relationship improbable and in which other factors suggesting an alternative etiology exist. Such factors include a known relationship of the adverse event to concomitant drug, the subject’s disease state, or environmental factors including common infectious diseases. |
| **Unrelated** | A clinical event, including a laboratory test abnormality, in which a relationship to the study drug seems improbable because of factors such as inconsistency with known effects of the study drug; lack of a temporal association with study drug administration; lack of association of the event with study drug withdrawal or rechallenge; and/or presence of alternative explanations for the event. Alternative explanations might include a known relationship of the adverse event to a concomitant drug, past medical history of a similar event, the subject’s disease state, or environmental factors. |
|  | |

## Follow-up of Unresolved Adverse Events

All adverse events should be followed until they are resolved, or the investigator assesses them as chronic or stable. The investigator should use his/her discretion in ordering additional tests as necessary to monitor the resolution of such events. A subject withdrawn from the study because of an adverse event must be followed by the investigator until the clinical outcome from the adverse event is determined. All follow-up information pertaining to serious adverse events must be forwarded to PTC Therapeutics within 24 hours of receipt (see Section 8.4). In the immediate post-treatment period, subjects must be followed for 28 days after the last dose of study treatment, or until all drug-related adverse events and ongoing serious adverse events have resolved or become stable, whichever is later. Long-term patient follow-up is described in Section 6.2.20.

# Withdrawal of subjects From Study Participation

Since this is a trial of relatively short‑term treatment, all subjects who receive study drug should remain in the study whenever possible. However:

- Any subject has the right to withdraw from the study at any time.
- Any subject whose condition substantially changes after entering the study should be carefully evaluated by the investigator in consultation with the PTC Therapeutics medical monitor. Such subjects should be withdrawn from study treatment if continuing would place them at risk.
- Any subject who becomes significantly noncompliant with study drug administration, study procedures, or study requirements should be withdrawn from study treatment when the circumstances increase risk to the subject or substantially compromise the interpretation of study results.
- Any subject who is unable to tolerate the 4-, 4-, and 8-mg/kg dose level of PTC124 should be withdrawn from study treatment.
- The investigator, in consultation with the PTC Therapeutics medical monitor, may withdraw any subject from the study treatment, if, in the investigator’s opinion, it is not in the subject’s best interest to continue.

The date the subject is withdrawn from the study and the reason for discontinuation will be recorded in the source documents and on the electronic data entry forms (see Section 10.4).

When a subject is withdrawn from the study (regardless of the reason), the investigator should encourage that all of the evaluations required at the Day 28 study visit be performed and that any additional evaluations be completed that may be necessary to ensure that the subject is free of untoward effects. The subject should be encouraged to seek appropriate follow‑up for any continuing health problems.

A subject who withdraws prematurely will be replaced if, based on a review of the available safety for that dose level, the investigator and the PTC Therapeutics medical monitor agree that the events leading to subject withdrawal are unlikely to constitute a safety risk for further subject enrollment and dose escalation. An additional subject can be assigned to study drug administration as described in Section 4.

A patient who completes the initial portion of the study (through the Day 56 visit) and is then lost to follow-up during the following 5-year long-term follow-up period will not be considered to have prematurely withdrawn.

# Statistics and Data Management

## Basis for Dose Escalation (4-, 4-, and 8-mg/kg to 10-, 10-, and 20-mg/kg)

Six subjects will be treated at the 4-, 4- and 8-mg/kg dose level. With this number of subjects, the probability of escalating to the 10-, 10- and 20-mg/kg dose level, based on the true rate of clinically significant adverse events in this study (see Section 5.3) at the 4-, 4- and 8-mg/kg dose, is given below:

| Table 9. Statistical Basis for Dose Escalation | |
| --- | --- |
| **True Incidence of Clinically Significant Adverse Events** | **Probability of Escalating** |
| 10% | 0.89 |
| 20% | 0.66 |
| 30% | 0.42 |
| 40% | 0.23 |
| 50% | 0.11 |
| 60% | 0.04 |
|  | |

Thus, if the true underlying proportion of clinically significant adverse events is 20% at the 4-, 4- and 8-mg/kg dose level, there is a substantial likelihood (66%) of escalating to the 10-, 10- and 20-mg/kg dose level. Conversely, if the true underlying proportion of clinically significant adverse events is 50% at the 4-, 4- and 8-mg/kg dose level, there is only an 11% chance of escalating to the 10-, 10- and 20-mg/kg dose level.

## Basis for Dose Escalation (10-, 10-, and 20-mg/kg to 20-, 20-, and 40-mg/kg)

Interim data from the 20 children with DMD completing 28 days of treatment at the 10-, 10-, and 20-mg/kg dose level indicate that PTC124 is safe. However, the 10-, 10-, and 20-mg/kg dose tested in these patients did not produce the target plasma concentrations known to be maximally active in preclinical animal models. Based on PK modeling, a dose level of 20-, 20-, and 40-mg/kg is projected to achieve the target plasma concentrations and to be associated with plasma exposures known to be safe in healthy volunteers and patients with CF. Given the substantial safety of PTC124 at lower dose levels and the current understanding of variability observed in the existing PK data, enrollment of 6 to 12 additional patients should be sufficient to assess the hypothesis that the 20-, 20-, 40- dose level will safely achieve the desired plasma concentrations.

## Power Calculation Based on Proposed Sample Size at the 10-, 10-, and 20-mg/kg Dose Level

At the 10-, 10- and 20-mg/kg dose level, this trial can evaluate the effect of PTC124 on dystrophin response (increase in full-length dystrophin on post-treatment muscle biopsy as assessed by immunofluorescence), excluding an uninteresting dystrophin response rate of ≤10% in favor of a target response rate of ≥40%. At least 18 subjects will be treated at the this dose level. This sample size has a power of 0.90 to reject the null hypothesis of a <10% response proportion in favor of an alternative hypothesis of a 40% target response proportion at a 1-sided significance level of <0.05 (see Table 10 in Section 9.4).

## Analysis Plan

All subjects who receive ≥1 dose of PTC124 will be included in the analyses of compliance and safety. For efficacy parameters, evaluable populations of subjects will comprise all subjects who have sufficient baseline and on-study measurements to provide interpretable results for the test of interest. The following methods will be used in the analysis of study data:

- - - - Subject characteristics at study entry will be summarized with frequency tables for categorical variables, and with descriptive statistics such as the mean, standard deviation, median, and range, as appropriate, for quantitative variables.
      - The proportion (and 90% confidence intervals [CIs]) of subjects with an on-treatment dystrophin response (as defined in Section 1.3) will be computed for each PTC124 dose level.
      - Among the subjects treated at the 10-, 10- and 20-mg/kg PTC124 dose level, these values will be used to determine if a <10% dystrophin response rate can be excluded at this dose level. Table 10 below shows the exact 90% binomial CIs on the true response rate for a range of possible values for the observed response rate. Thus, if ≥5 of 18 subjects demonstrate a dystrophin response, a dystrophin response rate of <10% can be excluded with substantial certainty. Sarcoglycan and dystroglycan responses will be assessed in a similar manner.

| Table 10. Exact Binomial Confidence Intervals by Dystrophin Response Rate (10-, 10-, and 20-mg/kg dose only) | | | |  |
| --- | --- | --- | --- | --- |
| **N** | **Dystrophin Responses (n)** | Response Rate  (%) | **90%CI  (%)** |  |
| 18 | 3 | 17 | 5-38 | |
|  | 4 | 22 | 8-44 | |
|  | 5 | 28 | 12-50 | |
|  | 6 | 33 | 16-55 | |
|  | 7 | 39 | 20-61 | |
|  | 8 | 44 | 24-66 | |
|  | 9 | 50 | 29-71 | |
|  | 10 | 56 | 34-76 | |
|  | 11 | 61 | 39-80 | |
|  | 12 | 67 | 45-84 | |
| **Abbreviations:** CI = confidence interval | | | |  |
|  | | | |  |

- - - - Additional analysis will be performed to compute an overall response rate for the patients from all 3 dose levels combined, as well as for the patients who received either of the 2 lower doses (4-, 4-, and 8-mg/kg and 10-, 10-, and 20-mg/kg) or either of the 2 higher doses (10-, 10-, and 20-mg/kg and 20-, 20-, and 40-mg/kg). Table 11 below shows the exact 90% binomial CIs on the true response rate for a range of possible values for the observed response rate in the patients who received:
- Either of the 2 lower doses (4-, 4-, and 8-mg/kg and 10-, 10-, and 20-mg/kg) where n is known to be 26 (ie, it is known that 6 patients are enrolled at the low dose and 20 patients are enrolled at the middle dose)
- Either of the 2 higher doses (10-, 10-, and 20-mg/kg and 20-, 20-, and 40-mg/kg) where n is assumed to be 26 (ie, it is known that 20 patients are enrolled at the middle dose and it is assumed that 6 patients will be enrolled at the high dose)
- Any of the 3 dose levels (4-, 4-, and 8-mg/kg, 10-, 10-, and 20-mg/kg or 20-, 20-, and 40-mg/kg) where n is assumed to be 32 (ie, it is known that 6 patients are enrolled at the low dose, that 20 patients are enrolled at the middle dose, and it is assumed that 6 patients will be enrolled at the high dose)

| Table 11****. Exact Binomial Confidence Intervals**** ****by Dystrophin Response Rate**** | | | | | |
| --- | --- | --- | --- | --- | --- |
| **Dose levels 4-, 4-, and 8-mg/kg  and 10-, 10-, and 20-mg/kg**  **or**  **Dose levels 10-, 10-, and 20-mg/kg  and 20-, 20-, and 40-mg/kg** | | | **Dose levels 4-, 4-, and 8-mg/kg,  10-, 10-, and 20-mg/kg and  20-, 20-, and 40-mg/kg combined** | | |
| **(N=26)** | | | **(N=32)** | | |
| **Dystrophin Responses** | **Response Rate** | **90% CI** | **Dystrophin Responses** | **Response Rate** | **90% CI** |
| **(n)** | **(%)** | **(%)** | **(n)** | **(%)** | **(%)** |
| 6 | 23 | 11-41 | 6 | 19 | 9-34 |
| 7 | 27 | 13-45 | 7 | 22 | 11-37 |
| 8 | 31 | 16-49 | 8 | 25 | 13-41 |
| 9 | 35 | 19-53 | 9 | 28 | 16-44 |
| 10 | 38 | 23-56 | 10 | 31 | 18-47 |
| 11 | 42 | 26-60 | 11 | 34 | 21-50 |
| 12 | 46 | 29-64 | 12 | 37 | 23-54 |
| 13 | 50 | 33-67 | 13 | 41 | 26-57 |
| 14 | 54 | 36-71 | 14 | 44 | 29-60 |
| 15 | 58 | 40-74 | 15 | 47 | 32-63 |
| 16 | 62 | 44-77 | 16 | 50 | 34-66 |
| 17 | 65 | 47-81 | 17 | 53 | 37-68 |
| 18 | 69 | 51-84 | 18 | 56 | 40-71 |
| 19 | 73 | 55-87 | 19 | 59 | 43-74 |
| **Abbreviations:** CI = confidence interval | | | | | |
|  | | | | | |

- - - - Dystrophin, sarcoglycan, dystroglycan Western blotting results, and dystrophin mRNA data will be described.
      - Changes in serum CK values will be analyzed using a 1-way repeated-measures analysis of variance (ANOVA).
      - Pre-treatment, on-treatment, and post-treatment (follow-up) myometry test values (mean and the median of the 3 replications) and timed function test values will be compared by means of ANOVA and pairwise comparisons.
      - For each subject, compliance will be described in terms of the proportion of drug actually taken relative to the amount that should have been taken. The distribution of compliance proportions will be summarized in frequency distributions by dose level. Deviations from planned dosing will be described.
      - Frequencies of adverse events and laboratory abnormalities will be tabulated by MedDRA System Organ Class, Preferred Term, dose level, worst severity, timing, outcome of the event, relationship to study drug, and seriousness.
      - PK parameters will be calculated using both noncompartmental and compartmental methods. Dose proportionality of PK parameters will be determined by performing an ANOVA on dose-normalized parameters.
      - Dystrophin and dystrophin-associated protein results from the muscle and dermal fibroblast cultures will be described.
      - Correlations among outcome measures (eg, dystrophin response in vivo versus dystrophin response in vitro, myometry changes versus serum CK changes) and among outcomes measures and PK parameters and type of stop codon (UAA, UAG, or UGA) will be explored using regression models or other appropriate techniques.

## Data Management and Quality Assurance

The services of a clinical research organization (CRO), Innovative Analytics (Kalamazoo, MI), will be provided for clinical data management. Designated personnel at the investigational sites will enter patient data via a secure, Web-based server into a study-specific electronic database located at the Innovative Analytics site. During the data collection process, representatives of Innovative Analytics will use automated quality assurance programs to identify missing data, selected protocol violations, out-of-range data, and other data inconsistencies. Requests for data clarification or correction will be forwarded to the investigative site for resolution. Listings, tables, and Statistical Analysis System (SAS) datasets will be provided to PTC Therapeutics for review.

Quality assurance and quality control systems will be implemented and maintained according to written standard operating procedures generated by Innovative Analytics and PTC Therapeutics to ensure that the data are generated, recorded, and reported in compliance with the protocol, GCP, and applicable regulatory requirements. Quality control will be applied to each cycle of data handling to ensure that all data are reliable and have been processed correctly.

## Data Monitoring Committee

External oversight for this trial will be provided by a DMC. The DMC will consist of at least 2 physicians experienced in treating DMD and a biostatistician. The primary responsibility of the DMC is to protect the safety and welfare of subjects participating in this clinical trial and to ensure the integrity of the clinical trial.

Specifically, for this study, the DMC will be responsible for:

- Examining accumulated safety data, PK, and compliance data in order to make recommendations concerning continuation, termination, or modification of the trial based on the safety of the interventions under study
- Reviewing major study design modifications proposed by PTC Therapeutics or the investigators prior to implementation of those modifications
- Reviewing the general progress of the studies as regards accrual, protocol violations, and study conduct

The DMC will review the interim data from the study once a total of 6 subjects have been treated at the low dose level, and again when all a total of ≥ 6 subjects have been treated at the middle dose level once sufficient safety, efficacy, and PK data are available for review. The DMC may review the safety data at other times as warranted by emerging results. Based on review of the safety data, the DMC can recommend continuation of the study unchanged, study interruption, study termination, modification of the trial, or alteration in the DMC monitoring plan.

Further information regarding the DMC review process is provided in the DMC charter (see Study Manual).

## Statistical and Data Management Rationale

The proposed sample size has been established to have high power (90%) in excluding an uninteresting dystrophin response rate of 10% in favor of a target response rate of 40%. The use of the subjects as their own controls and omission of a contemporaneous untreated control group relies upon knowledge that a dystrophin response is highly unlikely to occur spontaneously or with any existing therapy (eg, corticosteroids). Such a design provides efficiency in trial conduct given the limited numbers of subjects with nonsense-mutation-mediated DMD. The target response rate of ≥40% has been selected with consideration for the dystrophin response treatment effect size presumed necessary to produce relevant changes in muscle function in a future Phase 3 study.

The proposed analytical techniques are standard and appropriate for the types of data to be assessed. Similarly, the data management methods are appropriate to assure the quality and validity of the data derived from the study. Oversight of the trial by a DMC is appropriate to provide independent review of subject safety and study conduct.

# Obligations of the Investigator and the Sponsor

## Compliance with Ethical and Regulatory Guidelines

The investigator is responsible for ensuring that the clinical study is performed in accordance with the Declaration of Helsinki (revised version of Edinburgh, Scotland, 2000; see Appendix A), FDA GCP regulations (CFR 21 parts 50, 56, and 312), and the International Conference on Harmonisation (ICH) GCP guidance documents.

## Institutional Review Board

Prior to enrollment of subjects into the study, as required by the FDA and other regulatory authorities, the protocol and informed consent document will be reviewed and approved by an appropriate IRB. By signing the Statement of Investigator Form (FDA Form 1572), the investigator assures that approval of the trial protocol will be obtained from the IRB and that all aspects of the IRB review will be conducted in accordance with current regulations. Amendments to the protocol will be subject to the same IRB review requirements as the original protocol. Only changes necessary to eliminate apparent immediate hazards to the subjects may be initiated prior to IRB approval. In that event, the investigator must notify the IRB and PTC Therapeutics in writing within 5 working days after implementation. The investigator will also promptly notify the IRB of any serious, unexpected adverse events, or any other information that may affect the safe use of the drug during the course of the trial.

A letter documenting the IRB approval and a list of the names and titles of the IRB members must be received by PTC Therapeutics prior to the initiation of the study. All correspondence with the IRB should be retained in the investigator’s study file.

The investigator shall submit a progress report, at least once yearly, to the IRB and to PTC Therapeutics. As soon as possible after completion or termination of the study, the investigator will submit a final report to the IRB and to PTC Therapeutics. This report should include the dates of initiation and completion of the trial, a description of any changes in study procedures or amendments to the protocol, deviations from the protocol, the number and type of subjects evaluated, the number of subjects who discontinued (and the reasons for discontinuation), the number of subjects who completed the trial, and the results of the trial, including a description of any adverse events.

## Informed Consent/Assent

By signing the Statement of Investigator (FDA Form 1572), the investigator assures that informed consent/assent will be obtained from each subject or parent/guardian prior to study entry and that the informed consent/assent will be obtained in accordance with current regulations. The investigator will give each subject or parent/guardian full and adequate verbal and written information regarding the objective and procedures of the trial and the possible risks involved. An informed consent/assent document will be provided to each subject or parent/guardian in a language in which the subject or parent/guardian is fluent. This information must be provided to the subject or parent/guardian prior to undertaking any trial-related procedure. The written subject information must not be changed without prior approval by PTC Therapeutics and the IRB. Each subject or parent/guardian will be given a copy of the signed consent/assent form. The original signed informed consent forms will be retained by the investigator with the study records.

## Electronic Case Report Forms

An electronic case report form (CRF) is required and must be completed for each enrolled subject, with all required study data accurately recorded such that the information matches the data contained in medical records (eg, physician’s note, nurse notes, clinic charts and other study-specific source documents). These electronic data entry forms will be completed at each site by the investigator or investigator’s designee (eg, research coordinator). The electronic data entry forms will be submitted via a Web-based data capture system to Innovative Analytics, the data management CRO for this study. Any change of data will be made via the Web-based data capture system, with all changes tracked by the electronic system to provide an audit trail.

It is the investigator’s responsibility to ensure completion of data entry, and to review and approve all data entered into the electronic data capture system from the investigator’s site. A printed summary of the data entered into the electronic data entry forms for each subject, including any updates to the data for that subject, will be provided to the investigative site for review. This printed data summary of the electronically entered data for each subject must be signed by the principal investigator listed on the FDA Form1572. This signature serves to attest that the information contained on the electronic data entry forms is true. Whiting out and erasing on the printed version of the signed data summary document are not permitted. At all times, the investigator has final responsibility for the accuracy and authenticity of all clinical and laboratory data reported to PTC Therapeutics from the investigator’s site.

The printed, signed copies of the data summary documents for each subject will be retained by the investigator at the study site, with photocopies submitted to PTC Therapeutics upon request. The printed data summary documents, the data entered into the electronic data entry forms (electronic equivalents of CRFs), and the data contained therein are the sole property of PTC Therapeutics and should not be made available in any form to third parties, except for authorized representatives of PTC Therapeutics and of appropriate regulatory authorities, without written permission from PTC Therapeutics.

## Study Records

During the study, the investigator will maintain adequate records for the study, including medical records, source document records detailing the progress of the study for each subject, laboratory reports, printed and signed copies of the data summary documents of the data that has been captured in the electronic database for each subject (electronic equivalents of CRFs), signed informed consent forms, study drug disposition records, correspondence with the IRB, adverse event reports, and information regarding subject discontinuation and completion of the study. Current regulations require PTC Therapeutics (or an authorized designee) to inspect all documents and records required to be maintained by the investigator, including but not limited to medical records (office, clinic, or hospital) for the subjects enrolled in this trial. These regulations also allow the same records to be inspected by authorized representatives of the FDA or other regulatory authorities.

## Confidentiality

The names and identities of all research subjects will be kept in strict confidence and will not appear on electronic data entry forms or other records provided to or retained by PTC Therapeutics (or its authorized designee). The names and identities of the subjects need not be divulged; however, the records must nevertheless be inspected. This will be accomplished by blacking out the subject’s name and replacing the name with the subject’s study identification number on any record provided to or retained by PTC Therapeutics. The informed consent form must include appropriate statements explaining these requirements.

Attention is drawn to the regulations promulgated by the FDA under the Freedom of Information Act providing, in part, that information furnished to clinical investigators and the IRB will be kept confidential by the FDA only if maintained in confidence by the clinical investigator and the IRB. By signing this protocol, the investigator affirms to PTC Therapeutics that the investigator will maintain, in confidence, information furnished by PTC Therapeutics and will divulge such information to the IRB under an appropriate understanding of confidentiality with such board.

## Retention of Records

To enable evaluations and/or audits from regulatory authorities or PTC Therapeutics, the investigator agrees to keep records, including the identity of all participating subjects (sufficient information to link electronic data entry forms, and clinic records), all original signed informed consent forms, signed copies of the data summary documents of the data that has been captured in the electronic database for each subject (electronic equivalents of CRFs), and detailed records of study drug disposition. All records and documents pertaining to the study (including but not limited to those outlined in Section 10.5 above) will be maintained by the investigator until notification is received from PTC Therapeutics that the records no longer need to be retained. This will be for a period of at least a) 2 years after approval of the drug in the US and other countries; b) 5 years after non-approval of the New Drug Application (NDA) in the US or a similar regulatory submission in other countries; or c) 2 years after withdrawal of the US IND or a similar regulatory permission in other countries under which this study was conducted.

The investigator must obtain PTC Therapeutics’ written permission before disposing of any records. In order to avoid any possible errors, the investigator will contact PTC Therapeutics prior to the destruction of any study records. The investigator will promptly notify PTC Therapeutics in the event of accidental loss or destruction of any study records. If the investigator relocates, retires, or for any reason withdraws from the study, the study records may be transferred to an acceptable designee, such as another investigator, another institution, or to PTC Therapeutics.

## Monitoring and Auditing

In accordance with 21 CFR 312.56 and/or relevant ICH guidelines, PTC Therapeutics or a designee will periodically inspect all electronic data entry forms (see Section 10.4), study documents, research facilities, and clinical laboratory facilities associated with this study at mutually convenient times during and after completion of the study. As required by Subpart D of the IND regulations (Responsibilities of Sponsors and Investigators), the monitoring visits provide PTC Therapeutics with the opportunity to evaluate the progress of the study; verify the accuracy and completeness of data in the electronic data entry forms (100% check of data versus source documents); ensure that all protocol requirements, applicable FDA and other relevant regulations, and investigator’s obligations are being fulfilled; and resolve any inconsistencies in the study records. This includes inspection of all documents and records required to be maintained by the investigator, including but not limited to medical records (office, clinic, or hospital) for the subjects in this trial. The names and identities of all research subjects will be kept in strict confidence and will not appear on electronic data entry forms or other records provided to or retained by PTC Therapeutics. The investigator/institution guarantees direct access to source documents by PTC Therapeutics and appropriate regulatory authorities.

The trial site may also be subject to review by the IRB, to quality assurance audits performed by PTC Therapeutics, and/or to inspection by the FDA and/or other regulatory authorities. The IND regulations also require the investigator to allow authorized representatives of the FDA to inspect and make copies of the same records.

It is important that the investigator and relevant institutional personnel are available during the monitoring visits and possible audits or inspections and that sufficient time is devoted to the process.

## Termination of the Study

PTC Therapeutics reserves the right to discontinue the trial prior to inclusion of the intended number of subjects, but intends only to exercise this right for valid scientific or administrative reasons. The investigator, after consultation with the PTC Therapeutics medical monitor, reserves the right to discontinue the study for safety reasons at any time. After such a decision is taken, the investigator must contact all subjects who are continuing their participation in the study and must do so within a time period set by PTC Therapeutics. As directed by PTC Therapeutics, all study materials must be collected and all electronic data entry forms completed to the greatest extent possible.

## Dissemination of Results

The information developed during the conduct of this clinical study is considered confidential by PTC Therapeutics. This information may be disclosed as deemed necessary by PTC Therapeutics.

To allow for the use of the information derived from this clinical study and to insure compliance with current regulations, the investigator is obliged to provide PTC Therapeutics with complete test results and all data developed in this study. The information obtained during this study may be made available to other physicians who are conducting similar studies and to the FDA or other regulatory authorities.

PTC Therapeutics intends that the data from this study will be presented and published. The PTC Therapeutics staff under the direction of the PTC Therapeutics chief medical officer in collaboration with the investigator will be responsible for writing presentations and manuscripts for publication. Investigators will not be allowed to publish or present the data from this study without prior agreement with PTC Therapeutics.

# Bibliography

| 1. Barton-Davis ER, Cordier L, Shoturma DI, Leland SE, Sweeney HL. Aminoglycoside antibiotics restore dystrophin function to skeletal muscles of *mdx* mice. J Clin Invest. 1999 Aug;104(4):375-81. |
| --- |
| 1. Barton E, Zadel M, Welch EM, Trotta C, Paushkin S, Zhuo J, Tomizawa Y, Weetall M, Northcutt V, Babiak J, Miller LL, Sweeney L. PTC124 Nonsense Mutation Suppression Therapy of Duchenne Muscular Dystrophy. Neurology 2005;Vol 64 (6)Suppl1:A176 (abst # 060). |
| 1. Beenakker EA, van der Hoeven JH, Fock JM, Maurits NM. Reference values of maximum isometric muscle force obtained in 270 children aged 4-16 years by hand-held dynamometry. Neuromuscul Disord. 2001 Jul;11(5):441-6. |
| 1. Brussock CM, Haley SM, Munsat TL, Bernhardt DB. Measurement of isometric force in children with and without Duchenne's muscular dystrophy. Phys Ther. 1992 Feb;72(2):105-14. |
| 1. Drachman DB, Toyka KV, Myer E. Prednisone in Duchenne muscular dystrophy. Lancet 1974 Dec 14;2(7894):1409-12. |
| 1. Florence JM, Pandya S, King WM, Robison JD, Baty J, Miller JP, Schierbecker J, Signore LC. Intrarater reliability of manual muscle test (Medical Research Council scale) grades in Duchenne's muscular dystrophy. Phys Ther. 1992 Feb;72(2):115-22; discussion 122-6. |
| 1. Furlong MA, Fanburg-Smith JC, Miettinen M. The morphologic spectrum of hibernoma: a clinicopathologic study of 170 cases. Am J Surg Pathol. 2001 Jun;25(6):809-14. |
| 1. Griggs RC, Moxley RT 3rd, Mendell JR, Fenichel GM, Brooke MH, Pestronk A, Miller JP. Prednisone in Duchenne dystrophy. A randomized, controlled trial defining the time course and dose response. Clinical Investigation of Duchenne Dystrophy Group. Arch Neurol. 1991 Apr; 48(4): 383-8. |
| 1. Hunt SA, Baker DW, Chin MH, et al: ACC/AHA guidelines for the evaluation and management of chronic heart failure in the adult: executive summary. A report of the American College of Cardiology/American Heart Association Task Force on Practice Guidelines (Committee to revise the 1995 Guidelines for the Evaluation and Management of Heart Failure). J Am Coll Cardiol 2001 Dec;38:2101-13. |
| 1. Hyde SA, Steffensen BF, Floytrup I, Glent S, Kroksmark AK, Salling B, Werlauff U, Erlandsen M. Longitudinal data analysis: an application to construction of a natural history profile of Duchenne muscular dystrophy. Neuromuscul Disord. 2001 Mar;11(2): 165-70. |
| 1. McDonald CM, Abresch RT, Carter GT, Fowler WM Jr, Johnson ER, Kilmer DD, Sigford BJ. Profiles of neuromuscular diseases. Duchenne muscular dystrophy. Am J Phys Med Rehabil. 1995 Sep-Oct;74(5 Suppl):S70-92. |
| 1. Mendell JR, Moxley RT, Griggs RC. Randomized, double-blind six-month trial of prednisone in Duchenne's muscular dystrophy. N Engl J Med 1989 Jun; 320(24): 1592-7. |
| 1. Ohlendieck K, Campbell KP. Dystrophin-associated proteins are greatly reduced in skeletal muscle from *mdx* mice. J. Cell Bio. 1991;115:1685-1694 |
| 1. Politano L, Nigro G, Nigro V, Piluso G, Papparella S, Paciello O, Comi LI. Gentamicin administration in Duchenne patients with premature stop codon. Preliminary results. Acta Myol. 2003 May;22(1):15-21. |
| 1. Reitter B. Deflazacort vs. prednisone in Duchenne muscular dystrophy: trends of an ongoing study. Brain Dev. 1995; 17 Suppl:39-43. |
| 1. Sachs AB, Varani G. Eukaryotic translation initiation: there are (at least) two sides to every story. Nat Struct Biol. 2000 May;7(5):356-61. |
| 1. Simonds AK, Muntoni F, Heather S, Fielding S. Impact of nasal ventilation on survival in hypercapnic Duchenne muscular dystrophy. Thorax. 1998 Nov;53(11):949-52. Comment in: Thorax. 1999 Jun;54(6):564. |
| 1. Stedman H, Mendell J, Wilson JM, Finkel R, Kleckner AL. Phase I clinical trial utilizing gene therapy for Limb Girdle muscular dystrophy: α-, β-, γ-, or Δ-sarcoglycan gene delivered with intramuscular instillations of adeno-associated vectors. Human Gene Therapy, 2000 March;11:777–90. |
| 1. Stuberg WA, Metcalf WK. Reliability of quantitative muscle testing in healthy children and in children with Duchenne muscular dystrophy using a hand-held dynamometer. Phys Ther. 1988 Jun;68(6):977-82. |
| 1. Wagner KR, Hamed S, Hadley DW, Gropman AL, Burstein AH, Escolar DM, Hoffman EP, Fischbeck KH. Gentamicin treatment of Duchenne and Becker muscular dystrophy due to nonsense mutations. Ann Neurol. 2001 Jun;49(6):706-11. |
| 1. Wang D, Valdez MR, McAnally J, Richardson J, Olson E. The *Mef2c* gene is a direct transcriptional target of myogenic bHLH and MEF2 proteins during skeletal muscle development. Development 2001;128: 4623-33 |
| 1. Wilschanski M, Yahav Y, Yaacov Y, Blau H, Bentur L, Rivlin J, Aviram M, Bdolah-Abram T, Bebok Z, Shushi L, Kerem B, Kerem E. Gentamicin-induced correction of CFTR function in patients with cystic fibrosis and CFTR stop mutations. N Engl J Med. 2003 Oct 9;349(15):1433-41. |
| 1. Worton RG, Molnar MJ, Brais B and Karpati G. The muscular dystrophies. In: Scriver CR , Beaudet AL, Sly WS, Valle D, eds. The metabolic and molecular basis of inherited disease. 8th ed. Vol. 4. New York: McGraw-Hill, 2001:5493-523. |
