# Supplementary material for: Phase 2a Study of Ataluren-Mediated Dystrophin Production in Patients with Nonsense Mutation Duchenne Muscular Dystrophy
Source: PLoS One. 2013 Dec 11;8(12):e81302. doi: 10.1371/journal.pone.0081302 (PMC3859499; doi:10.1371/journal.pone.0081302)
Supplement: Appendix S1 — Supplemental Appendix. (DOC) [file pone.0081302.s004.doc]

**Supplemental Appendix**

*Inclusion Criteria*

Patients who met all of the following conditions were eligible for enrollment into the study:

1. Diagnosis of DMD based on a clinical phenotype that had presented by 5 years of age, with increased serum CK levels and diminished staining for dystrophin on a muscle biopsy (diminished sarcolemmal staining with an antibody to the C-terminal portion of the dystrophin protein). For patients who had not previously had a muscle biopsy, a biopsy from a muscle other than the EDB muscle or the baseline EDB muscle biopsy could be used to establish study eligibility.
2. Presence of a nonsense mutation in the dystrophin gene.
3. Documentation that dystrophin gene sequencing had been performed by the University of Utah (Salt Lake City, Utah, USA) or, if sequencing had not previously been performed by the University of Utah, that a blood sample had been sent to the University of Utah for the confirmatory dystrophin gene sequencing. Patients who had already had gene sequencing performed at the University of Utah were not required to have a repeat test done. Patients who had documentation of a nonsense mutation by other methods were required to provide a blood sample for gene sequencing but were not required to wait for confirmatory results from the University of Utah to start study therapy.
4. Physical examination or radiographic imaging evidence of EDB muscles in both feet. Patients who did not have physically palpable EDB muscles were to undergo an ultrasound or magnetic resonance imaging (MRI) of the feet for documentation of the bilateral presence of EDB muscles that had not undergone fibrosis. A biopsy of the TA muscle was to be obtained from patients who did not have EDB muscle available for biopsy.
5. Ability to ambulate or, if nonambulatory, no requirement for ventilator support.
6. Male sex.
7. Age ≥5 years.
8. If known to be sexually active, willingness to abstain from sexual intercourse or to employ a barrier or medical method of contraception during the 28‑day ataluren treatment period and the 28‑day posttreatment follow‑up period.
9. Willingness and ability to comply with the scheduled study visits, the drug administration plan, laboratory tests, study restrictions, study procedures (including muscle biopsies and pharmacokinetic sampling), and timed function tests and myometry testing (for patients who were able to perform such testing).
10. Ability to provide written informed consent if ≥18 years of age or written informed assent (with parental/guardian consent) if ≥7 years of age but <18 years. Consent of the parent or legal guardian alone was obtained for patients who were <7 years of age.
11. Documentation of a personally signed and dated informed consent document (assent was also required for children ≥7 years but <18 years of age) indicating that the patient, parent, or legal guardian had been informed of all pertinent aspects of the trial.

*Exclusion Criteria*

Patients were excluded from the study for any of the following reasons:

1. Prior or ongoing medical condition (eg, concomitant illness, psychiatric condition, alcoholism, drug abuse), medical history, physical findings, ECG findings, or laboratory abnormality that, in the investigator’s opinion, could have adversely affected the safety of the patient, made it unlikely that the course of treatment or follow-up would be completed, or impaired the assessment of study results.
2. Clinical symptoms and signs of congestive cardiac failure (American College of Cardiology/American Heart Association Stage C or Stage D [1]).
3. Positive hepatitis B surface antigen, hepatitis C antibody test, or human immunodeficiency virus (HIV) test.
4. Hemoglobin <10 g/dL.
5. Serum albumin <2·5 g/dL.
6. Abnormal gamma-glutamyl transferase (GGT) or total bilirubin (above upper limit of normal [ULN] based on the laboratory’s reference range).
7. Abnormal renal function (serum creatinine >1·5 times the ULN based on the laboratory’s reference range).
8. History of solid organ or hematological transplantation.
9. Ongoing immunosuppressive therapy with agents other than corticosteroids.
10. Exposure to another investigational drug within 28 days before the start of study treatment.
11. Ongoing participation in any other therapeutic clinical trial at the time of enrollment in this study.
12. Ongoing use of thiazolidinedione peroxisome proliferator-activated receptor gamma (PPAR-γ) agonists, eg, rosiglitazone (Avandia or equivalent) or pioglitazone (Actos or equivalent).
13. Change in systemic corticosteroid therapy (eg, initiation of treatment; cessation of treatment; or change in dose, schedule, or type of steroid) within 3 months before the start of study treatment.
14. Treatment with systemic aminoglycoside antibiotics within 4 weeks before the start of study treatment. Patients were allowed to receive systemic antibiotics as clinically necessary for life-threatening infections during the study; however, use of aminoglycoside antibiotics was to be avoided if possible.

*Assessment of In Vivo Dystrophin Expression*

Evaluation of the in vivo dystrophin response to ataluren treatment was performed in the laboratory of Carsten G. Bönnemann, MD, at the Children’s Hospital of Philadelphia (Philadelphia, Pennsylvania, USA). At the time of assay, a portion of the muscle was cryosectioned into 9-micron slices and serially double-stained using monoclonal spectrin (Spectrin AB, Novacastra Laboratories, Newcastle, UK) and dystrophin (DYS2 neat, BioGenex Laboratories, San Ramon, USA) antibodies. The sections were first stained with DYS2, followed by staining with goat antimouse AlexaFluor-488 (Molecular Probes, Eugene, Oregon, USA). The sections were then fixed for 1 minute in 1% paraformaldehyde before staining with a second primary antibody (Spectrin AB, Novacastra Laboratories, Newcastle, UK ), followed by staining with goat antimouse Zenon AlexaFluor-568 (Invitrogen Corporation, Carlsbad, USA). Optimization studies showed no evidence for secondary antibody cross-staining with the use of the intervening paraformaldehyde fixation step.

Images were obtained on an Olympus IX70 confocal microscope. Fields for fluorescent imaging were randomly selected under bright field microscopy (either 4 or 5 fields per patient sample, whether pre- or posttreatment), and 12-bit fluorescent images were then obtained. All of the images that were to be analyzed for a given patient (whether pre- or posttreatment) were photographed under the same laser intensity, aperture, and photomultiplier tube sensitivity settings.

For the qualitative analysis, three expert reviewers at Children’s Hospital of Philadelphia who were blinded to timepoint (ie, pretreatment or posttreatment) and dose level independently compared dystrophin expression in the baseline and posttreatment immunostaining images. If ≥2/3 blinded reviewers observed more dystrophin staining in a subject’s posttreatment specimen compared to his pretreatment specimen, that subject was considered a responder. See Figure 2 for an example of responder images; see Figure S1 for an example of non-responder images.

For the quantitative analysis, the images obtained at the Children’s Hospital of Philadelphia were analyzed using a custom MetaMorph (Molecular Devices, Inc, Union City, California, USA) script in the laboratory of Kevin M. Flanigan, MD at the University of Utah Hospital (Salt Lake City, Utah, USA). The regions of interest in the spectrin image were selected using an intensity threshold that was chosen by the user. The regions of interest were then converted into a binary mask in which all areas of interest had a pixel intensity value of 1, and the background had a pixel intensity value of 0. Spectrin areas smaller than 3-by-3 pixels were removed from the image by an erosion step designed to eliminate background noise. The image was then dilated, restoring contiguous regions, which were defined as spectrin-positive regions. In order to assess dystrophin signal only at the sarcolemmal membrane, dystrophin intensity was evaluated only at these spectrin-positive regions. The intensity of the dystrophin staining in spectrin positive areas was then scored as to intensity on an arbitrary scale from 0 to 4096. The maximum, 4096, was determined by the 12-bit information content of the image. The presumed mechanism of action of ataluren would be expected to result in a general increase in dystrophin expression, not in an increase in the number of dystrophin-positive (revertant) fibers. Although present in muscle biopsies from many patients with DMD due to somatic reversion or alternative splicing [2-4] revertant fibers appear to have no correlation with patients’ functional ability.[5] Furthermore, myocytes with revertant fibers are randomly distributed through many biopsies from patients with DMD, and their inclusion in this quantitative analysis could have resulted in a false-positive or false-negative result. Therefore, revertant fibers, which were arbitrarily defined as those that showed a dystrophin signal of greater than 30% of the maximum intensity (>1228 out of 4096 on the intensity scale), were excluded from this analysis. The MetaMorph script excluded any dystrophin-positive pixel that met this threshold and that was found in the spectrin-positive regions, thus eliminating revertant fibers from the analysis. Dystrophin intensity values for all spectrin-positive regions were averaged, thereby generating an average dystrophin value for the entire image.

*Assessment of In Vitro Dystrophin Expression*

In vitro culture of myotubes from the patient muscle biopsies was performed in the laboratory of H. Lee Sweeney, PhD (University of Pennsylvania, Philadelphia, Pennsylvania, USA). For these assessments, primary muscle cells were derived from the muscle biopsy specimens that were obtained before the start of ataluren treatment. Human satellite cells were then obtained and frozen for subsequent culture experiments using explant culture technique.[6] Briefly, sterile biopsies were finely minced into small fragments and equilibrated in medium containing 90% fetal calf serum (FCS; Invitrogen, Carlsbad, CA) and 10% dimethyl sulfoxide (DMSO) and then frozen and stored in liquid nitrogen. For experiments, the frozen fragments were thawed, rinsed with Dulbecco's modified Eagle's medium (DMEM-glutamax; Invitrogen, Carlsbad, CA) and plated onto Petri dishes in DMEM supplemented with 20% FCS. Myoblasts obtained from the explant cultures were expanded for 2 passages, and then frozen for future use.

Myoblast cultures were initiated by plating the thawed frozen cells on collagen-coated Aclar coverslips (Electron Microscopy Sciences, Fort Washington, PA) in growth medium that contained 20% fetal bovine serum (Thermo Scientific Hyclone, Logan, UT) and 1% penicillin-streptomycin in Ham's F10 culture media (Invitrogen, Carlsbad, CA).

After reaching 80% confluence, the cells were then switched to differentiation medium, which contained 97% Dulbecco’s modified Eagle media (DMEM, Invitrogen, Carlsbad, CA), 0.001% insulin (Sigma-Aldrich, St. Louis, MO), 0.01% apo-transferrin (Sigma-Aldrich, St. Louis, MO), and 1% penicillin-streptomycin (Invitrogen, Carlsbad, CA). The growth medium or differentiation medium was changed with fresh medium every third day.

Differentiation of the myotubes was allowed to progress for 3 days before ataluren treatment was initiated. Ataluren (at concentrations including 10 μg/mL) was then added to the cultures in differentiation medium and the cultures were allowed to differentiate for an additional 9 days under constant ataluren treatment with a change of differentiation medium and ataluren every third day.

On the 12th day after switching to the differentiation medium (ninth day of ataluren treatment), the myotubes were fixed with 2% formaldehyde in PBS at room temperature for 3 min. After permeabilization by 0.5% Triton X-100, immunofluorescence staining was then performed by incubating the cells with a monoclonal anti-dystrophin antibody (DYS2, non-diluted; Biogenix, San Ramon, CA) and a polyclonal anti-spectrin antibody (ab11182, 1:40; Abcam, Cambridge, MA) for 90 min at 37°C. This was followed by incubation with secondary antibodies [Alexa Fluor 488 goat anti-mouse IgG1 (1:100; Invitrogen, Carlsbad, CA), or Alexa Fluor 568 goat anti-rabbit IgG (Invitrogen 1:100)] for 60 min at 37 °C. Finally, the cells on Aclar coverslips were mounted on a slide using CITIFLUOR (Electron Microscopy Sciences, Fort Washington, PA) for final imaging.

Confocal imaging was used for the quantification of dystrophin expression in ataluren-treated myotubes. To avoid cross-talk between channels, the immunostained myotubes were sequentially excited at 543nm, 488nm and 633nm and the sequential scanning images were observed via a Leica oil immersed 63X/1.4 objectives and digitally recorded with a Leica DMR/TCS SL confocal microscopy system equipped with a 65mW Argon laser, a 1.2mW GreNe laser and a 10mW HeNe laser. To ensure imaging consistency for all dose-response measurements, all confocal microscopy system parameter settings were the same except the photomultiplier tube (PMT) gain setting which had to be varied from patient to patient to realize the maximal dynamic range of PMT signal detection. For imaging each patient’s myotubes, the proper gain was chosen at the maximal dystrophin expression and the same gain was subsequently used for acquiring all images for that patient’s cultures. Thus, for each patient’s cultures, all confocal parameters were set exactly the same for all doses while, between patients cultures, confocal parameters were the same except the PMT gain settings. The images were recorded as 8-bit digital resolution and 1024x1024 scan format.

Dystrophin expression was evaluated by the quantification of immunofluorescence profiles of ataluren-treated and untreated myotubes. The fluorescence profiles were measured by using the quantification tools of the Leica Confocal Software (version 2.61, Build 1537). Line profiles and/or stack profiles were adopted to determine the mean amplitude of the region of interest (ROI) which, whenever possible, spanned the whole myotube (except nuclei). Spectrin staining of myotubes was used to define the edges of the myotubes. For each patient, the arithmetic mean of the mean amplitudes of all measured ROIs for each dose was adopted as the value responding to the dose. Since each patient was imaged at different PMT gain settings, all patient data were calculated to the same gain based on the PMT gain calibration curve before they were grouped for statistical analysis. A Perl script was written and used to automate the calculation and eliminate potential calculation mistakes.

*Statistical Power Calculation*

At the 10-, 10- and 20-mg/kg dose level, this trial could evaluate the effect of PTC124 on dystrophin response (increase in full-length dystrophin on post-treatment muscle biopsy as assessed by immunofluorescence), excluding a response rate of ≤10% in favor of a target response rate of ≥40%. At least 18 subjects were to be treated at the this dose level. This sample size has a power of 0.90 to reject the null hypothesis of a <10% response proportion in favor of an alternative hypothesis of a 40% target response proportion at a 1-sided significance level of <0.05.

**References**

1. Hunt SA, Baker DW, Chin MH, Cinquegrani MP, Feldman AM, et al. (2001) ACC/AHA guidelines for the evaluation and management of chronic heart failure in the adult: executive summary. A report of the American College of Cardiology/American Heart Association Task Force on Practice Guidelines (Committee to revise the 1995 Guidelines for the Evaluation and Management of Heart Failure). J Am Coll Cardiol 38:2101-2113.
2. Klein CJ, Coovert DD, Bulman DE, Ray PN, Mendell JR, Burghes AH. (1992) Somatic reversion/suppression in Duchenne muscular dystrophy (DMD): evidence supporting a frame-restoring mechanism in rare dystrophin-positive fibers. Am J Hum Genet 50(5): 950-959.
3. Lu QL, Morris GE, Wilton SD, Ly T, Artem'yeva OV, et al. (2000) Massive idiosyncratic exon skipping corrects the nonsense mutation in dystrophic mouse muscle and produces functional revertant fibers by clonal expansion. J Cell Biol 148(5):985-996.
4. Bertoni C, Lau C, Rando TA. (2003) Restoration of dystrophin expression in mdx muscle cells by chimeraplast-mediated exon skipping. Hum Mol Genet 12(10):1087-1099.
5. Arechavala-Gomeza V, Kinali M, Feng L, Guglieri M, Edge G, et al. (2010) Revertant fibres and dystrophin traces in Duchenne muscular dystrophy: implication for clinical trials. Neuromuscul Disord 20(5):295-301.
6. Decary S, Mouly V, Hamida CB, Sautet A, Barbet JP, Butler-Browne GS. (1997) Replicative potential and telomere length in human skeletal muscle: implications for satellite cell-mediated gene therapy. Hum Gene Ther 8(12):1429-1438.
